# A[A_6_Ch][Si_12_P_20_] (A = Sr, Ba; Ch = S, Se, Te): achieving a wide band gap in pnictides by constructing [A_6_Ch] octahedral ionic units

**DOI:** 10.1039/d6sc00313c

**Published:** 2026-05-13

**Authors:** Huikang Jiang, Guang Peng, Ning Ye, Jindong Chen

**Affiliations:** a State Key Laboratory of Crystal Materials, Tianjin Key Laboratory of Functional Crystal Materials, Institute of Functional Crystal, College of Materials Science and Engineering, Tianjin University of Technology Tianjin 300384 China nye@email.tjut.edu.cn cjd1225@email.tjut.edu.cn; b Tianjin Key Laboratory of Quantum Optics and Intelligent Photonics, School of Science, Tianjin University of Technology Tianjin 300384 China; c SEU-FEI Nano-Pico Center, Key Laboratory of MEMS of Ministry of Education, School of Integrated Circuit, Southeast University Nanjing 210096 China

## Abstract

The design and synthesis of novel inorganic pnictides has long been challenging due to the difficulty in achieving a wide band gap, with most pnictides exhibiting a narrow band gap *E*_g_ <2.0 eV. This work reports the first synthesis of A–M–Pn–Ch phase A[A_6_Ch][Si_12_P_20_] (A = Sr, Ba; Ch = S, Se, Te) by introducing highly electronegative chalcogen elements (S, Se, Te) into the Ba/Sr–Si–P system. They exhibit wide band gaps of 1.91–2.27 eV, significantly outperforming known compounds in the Ba/Sr–Si–P and A–M–Pn–X (X = halogen) systems. Theoretical calculations reveal that the wide band gaps originate from the electronic regulation effect of the [A_6_Ch] octahedral ionic units, whose moderate ionic-covalent hybrid bonding characteristics promote charge localization and effectively suppress the metallic behavior of the system. Moreover, by constructing the mixed octahedral ionic unit [Ba_2_Sr_4_Ch] as an interpenetrated guest, the inversion symmetry of the interpenetrated host [Si_12_P_20_] covalent framework is successfully broken, enabling Ba[Ba_2_Sr_4_Ch][Si_12_P_20_] to crystallize in the non-centrosymmetric space group *F4̄*3*m*. This work proposes a strategy based on regulating the electronic structure *via* [A_6_Ch] octahedral ionic units, providing a previously unreported paradigm for the design and synthesis of wide-band-gap pnictides.

## Abstract 2

First A–M–Pn–Ch pnictides A[A_6_Ch][Si_12_P_20_] (A = Sr, Ba; Ch = S, Se, Te) with interpenetrated topology show wide band gaps (1.91–2.27 eV). The wide gap arises from [A_6_Ch] octahedral units promoting charge localization and suppressing metallicity.

## Graphical abstract

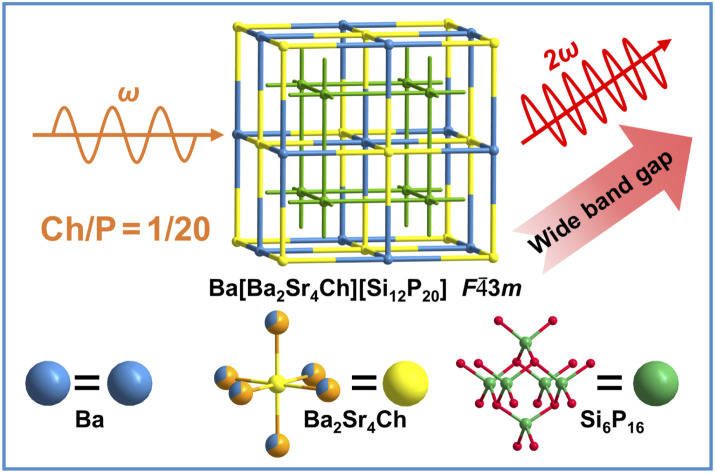

## Introduction

Inorganic pnictides with photovoltaic, superconductive, nonlinear optical (NLO), thermoelectric, radiation detection, and catalytic properties, among others, have attracted widespread interest and sustained attention.^1–6^ Pnictides are promising infrared NLO candidates due to their generally large NLO coefficients and wide IR transparency windows. Nevertheless, pnictides suffer from narrow band gaps (*E*_g_ < 2.0 eV for most pnictides).^7–12^ In oxides and chalcogenides,^13–17^ introducing strongly ionic, large-radius alkali metals (*e.g.* K, Rb, or Cs) or alkaline-earth metals (*e.g.* Ca, Sr, or Ba) is a common design strategy. These cationic species not only balance the charge of the anionic framework but also enhance the overall ionicity of the system and reduce interatomic orbital overlap, thereby widening the band gap. For instance, in the BaGa_4_S_7_ crystal, Ba^2+^ ions effectively tailor the covalent network of the GaS_4_ tetrahedral framework, increasing the system's ionicity. Consequently, BaGa_4_S_7_ exhibits a larger band gap (3.54 eV) compared to Ga_2_S_3_ (2.80 eV), AgGaS_2_ (2.76 eV), and ZnGa_2_S_4_ (3.18 eV).^18–20^ However, in IA/IIA-IIIA/IVA-P pnictides containing strongly ionic, large-radius alkali metals (*e.g.* K, Rb, or Cs) or alkaline-earth metals (*e.g.* Ca, Sr, or Ba), the band gaps are generally lower than those of SiP (1.71 eV), ZnSiP_2_ (2.26 eV), and ZnGeP_2_ (1.97 eV). Examples include BaSi_7_P_10_ (1.48 eV), SrSi_7_P_10_ (1.51 eV), Ba_2_SiP_4_ (1.45 eV), Sr_2_SiP_4_ (1.45 eV), Ba_2_Si_3_P_6_ (1.88 eV), and BaGe_2_P_2_ (1.32 eV).^21–24^ Although the introduction of ions such as Ba/Sr enhances local ionicity, the size effect and electrostatic interactions of these ions typically reduce the coordination number of P/As atoms in the system (to less than 3CN). Due to their weak electron affinity and insufficient electronegativity, P/As atoms cannot stabilize a formal eight-electron closed-shell configuration, leading to the delocalization of non-bonding electron pairs. As a result, the covalent interaction between IA/IIA and P disappears, and it tends toward an intermetallic interaction, causing the band gap to decrease rather than increase. Therefore, effectively widening the band gap of pnictides requires the comprehensive regulation of their “ionicity–covalency–metallicity”.

Although pnictides containing large-radius IA/IIA metals generally do not exhibit wide band gaps, this does not preclude their use as compositional elements when designing novel pnictide nonlinear optical materials. Our team pioneered the introduction of halogen X into the A–M–Pn system (*i.e.*, targeting the A–M–Pn–X phase field) to construct asymmetric A–X ionic units, breaking the local inversion symmetry of M–Pn groups and enabling the directional assembly of covalent groups.^25^ This approach successfully yielded the balanced-performance salt-including pnictides [Sr_4_Br]_2_[Mg_3_Si_25_P_40_] (5.2 × AGS, *E*_g_ = 1.9 eV) and [Ba_3_Br][GaSi_10_P_16_] (6.4 × AGS, *E*_g_ = 1.86 eV). The occupied np orbitals of halogens reside at lower energy levels, which can lower the energy level of the valence band maximum (VBM) of the system. Simultaneously, the formation of A–X ionic units weakens the contribution of the (*n*-1)d empty orbitals of IA/IIA metals to the conduction band minimum (CBM), thereby helping to elevate the CBM and effectively widen the band gap. The electronegativities of the chalcogen elements S (2.58) and Se (2.55) are greater than that of P (2.19). Therefore, introducing S and Se is expected to enhance the band gap of pnictides. Chalcogens tend to coordinate with A-site cations, collectively balancing the charge of the system, thereby forming chalcogenide-including pnictides. This involves introducing Ch into the A–M–Pn system (*i.e.*, targeting the A–M–Pn–Ch phase field) to construct asymmetric A–Ch ionic units that break the local inversion symmetry of M–Pn groups. Compared to A–X bonds, A–Ch bonds possess stronger covalent character, which helps reduce the overall ionicity and metallicity of the system, enabling band gap enlargement.

Guided by the above rationale, we successfully designed and synthesized eight pnictides: Ba[Ba_6_Ch][Si_12_P_20_] (Ch = S, Se, Te), Ba[Ba_2_Sr_4_Ch][Si_12_P_20_] (Ch = S, Se, Te), and Sr[Sr_6_Ch][Si_12_P_20_] (Ch = Se, Te). These compounds exhibit a unique interpenetrated topological structure. To date, there have been no reports of pnictides in the A–M–Pn–Ch phase field. The highly symmetric [Ba_6_Ch]/[Sr_6_Ch] octahedral ionic motifs force the (Si_6_P_16_) covalent units into an inversion–symmetric configuration, resulting in crystallization in the centrosymmetric space group *Fm*3̄*m*. The difference in ionic radii between Ba^2+^ (1.60 Å, CN = 12) and Sr^2+^ (1.44 Å, CN = 12) allows the construction of [Ba_2_Sr_4_Ch] mixed octahedrons, which reduces the symmetry of the interpenetrated guest ionic moiety. This effectively breaks the local inversion symmetry of the structure, leading to differentiated arrangements of the interpenetrated host covalent (Si_6_P_16_) building units, and increases the number of Wyckoff sites, leading to crystallization in the NCS space group *F4̄*3*m*. The Ba[Ba_2_Sr_4_Ch][Si_12_P_20_] series exhibits wide band gaps (2.08–2.15 eV, significantly superior to known compounds in the Ba/Sr–Si–P and A–M–Pn–X systems), moderate SHG responses (0.35–0.45 × AGS), and broad infrared transmission ranges (∼10.2 µm). From the perspective of the local coordination environments of both the interpenetrated guest octahedral ionic units [A_6_Ch] and the interpenetrated host covalent framework [Si_12_P_20_], this work provides an in-depth elucidation of the symmetry influence exerted by the [A_6_Ch] guest units on the host covalent structure and the associated band gap enhancement mechanism.

## Results and discussion

Ba[Ba_6_Ch][Si_12_P_20_] (Ch = S, Se, Te) and Sr[Sr_6_Ch][Si_12_P_20_] (Ch = Se, Te) are isostructural and crystallize in the centrosymmetric space group *Fm*3̄*m* (No. 225), with unit cell parameters of *a* = *b* = *c* = 15.530(15)–15.826(8) Å, *α* = *β* = *γ* = 90°, and *Z* = 4. The Ba[Ba_2_Sr_4_Ch][Si_12_P_20_] (Ch = S, Se, Te) compounds are also isostructural with each other, belonging to the non-centrosymmetric space group *F4̄*3*m* (No. 216), with unit cell parameters of *a* = *b* = *c* = 15.567(6)–15.6412(4) Å, *α* = *β* = *γ* = 90°, and *Z* = 4 (Tables S1–S5, SI). Ba[Ba_6_S][Si_12_P_20_] and Ba[Ba_2_Sr_4_S][Si_12_P_20_] were selected as representative structures for detailed analysis. The asymmetric unit of Ba[Ba_6_S][Si_12_P_20_] contains six crystallographically independent atoms: two Ba atoms, one S atom, one Si atom, and two P atoms. In contrast, the asymmetric unit of Ba[Ba_2_Sr_4_S][Si_12_P_20_] contains eight crystallographically independent atoms: one Ba atom, one Ba/Sr (1 : 2) mixed-occupancy atom, one S atom, two Si atoms, and three P atoms.

**Fig. 1:**
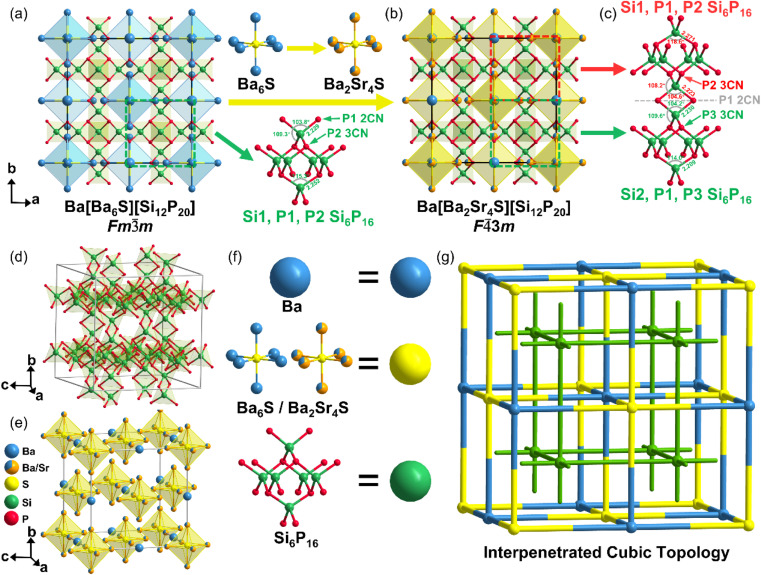
Crystal structure. (a) The crystal structure of Ba[Ba_6_S][Si_12_P_20_] within the unit cell. (b) The crystal structure of Ba[Ba_2_Sr_4_S][Si_12_P_20_] within the unit cell. (c) Two connected (Si_6_P_16_) units. (d) The host covalent framework of (Si_6_P_16_). (e) Guest ionic units of Ba and [Ba_6_S]/[Ba_2_Sr_4_S]. (f) A schematic diagram showing blue spheres representing Ba atoms, yellow spheres representing [Ba_6_S]/[Ba_2_Sr_4_S] units, and green spheres representing (Si_6_P_16_) units. (g) A schematic diagram of the interpenetrated topological structure.

The structure of Ba[Ba_6_S][Si_12_P_20_] consists of a three-dimensional covalent [Si_12_P_20_]^12−^ inorganic framework built from SiP_4_ tetrahedra, with Ba^2+^ cations and [Ba_6_S]^10+^ units acting as charge-balancing guests occupying the channels (Fig. 1a). The [Si_12_P_20_] framework is constructed from (Si_6_P_16_) units connected *via* edge-sharing SiP_4_ tetrahedra and can be described based on six vertex-sharing SiP_4_ tetrahedra (Fig. 1d). The edge-sharing P1 atoms are 2CN, while the vertex-sharing P2 atoms are 3CN. The Si1–P1 and Si1–P2 bond lengths are 2.229 Å and 2.252 Å, respectively, and the P1–Si1–P1, P1–Si1–P2, and P2–Si1–P2 bond angles are 103.8°, 109.3°, and 115.3°, respectively, indicating a certain degree of distortion in the SiP_4_ tetrahedra. The S atom coordinates with six neighboring Ba1 atoms to form a [Ba_6_S] octahedron and does not directly coordinate with Ba2 atoms. The [Ba_6_S] and Ba2 units act as ionic guests, alternately arranged within the [Si_12_P_20_] covalent framework (Fig. 1e), forming an interpenetrated cubic topology (Fig. 1f and g). Similar interpenetrated structures have been reported in MOF materials such as [Co_4_O(bpdc)_3_][Zn_4_O(L1)_3_] and the borate Na_4_[Al_3_(BO_3_)_4_]·Cl,^26–28^ but they are extremely rare in pnictide and chalcogenide systems.

The interpenetrated host covalent framework [Si_12_P_20_] possesses large pores, providing a structural basis for the substitution of ionic sublattices. Both Ba[Ba_6_Ch][Si_12_P_20_] (Ch = S, Se, Te) and Sr[Sr_6_Ch][Si_12_P_20_] (Ch = Se, Te) are centrosymmetric. Even when Ba is fully replaced by Sr, the [Ba_6_Ch] and [Sr_6_Ch] octahedra remain highly symmetric, resulting in the covalent (Si_6_P_16_) units adopting an inversion–symmetric configuration, similar to the case of Cs[Ba_6_Cl][Si_12_P_20_] reported by Kirill Kovnir. Our systematic investigation of six-coordinated [A_6_Ch]/[A_6_X] group compounds (Table S6) shows that most crystallize in centrosymmetric space groups,^29^ which is unfavorable for nonlinear optical crystals. Our team recently proposed a strategy using asymmetric ionic units to direct the orientation of covalent groups, which can effectively guide the formation of non-centrosymmetric structures. Although Ba and Sr are alkaline earth metals with very similar physicochemical properties and tend to result in mixed occupancy, the difference in their ionic radii (Ba^2+^: 1.60 Å, Sr^2+^: 1.44 Å, CN = 12) can reduce the symmetry of the ionic units.

Based on this strategy, we replaced the KI flux with SrBr_2_ and increased the reaction temperature from 900 °C to 1000 °C. Sr from the flux partially substituted the Ba1 site in [Ba_6_S], forming Ba[Ba_2_Sr_4_S][Si_12_P_20_] (Fig. 1b). In the resulting [Ba_2_Sr_4_S] mixed octahedral unit, S1 remains at the 4a site, while Ba1/Sr1 occupies the 24f site with mixed occupancy (Table 1). The mixed cation occupancy and atomic displacement directly reduce the symmetry, breaking the highly symmetric [Ba_6_S] octahedral configuration originally corresponding to Ba1 atoms at the 24e site. The symmetry breaking of the guest unit propagates through the lattice, perturbing the [Si_12_P_20_] covalent framework and causing the splitting of originally equivalent atomic sites: the Si atoms split from a single 48g site (Si1) into two independent 24g sites (Si1 and Si2); the 2CN P1 atoms shift from the 48i to the 48h site; and the 3CN P atoms split from the 32f site (P2) into two 16e sites (P2 and P3). The two distinct (Si_6_P_16_) structural units, composed of Si1–P1–P2 and Si2–P1–P3, exhibit differentiated distortion. For the Si1–P1–P2 unit, the Si1–P1 and Si1–P2 bond lengths are 2.223 Å and 2.271 Å, respectively, with P1–Si1–P1, P1–Si1–P2, and P2–Si1–P2 bond angles measuring 104.6°, 108.2°, and 118.6°, respectively. For the Si2–P1–P3 unit, the Si2–P1 and Si2–P3 bond lengths are 2.230 Å and 2.209 Å, respectively, with P1–Si2–P1, P1–Si2–P3, and P3–Si2–P3 bond angles measuring 104.2°, 109.6°, and 114.0°, respectively. The coexistence of these two (Si_6_P_16_) units with distinct distortion confirms the asymmetric deformation of the covalent framework and the deviation from a centrosymmetric nature (Fig. 1c). The observed SHG signals further verify the non-centrosymmetric nature of the Ba[Ba_2_Sr_4_Ch][Si_12_P_20_] (Ch = S, Se, Te) system, fully demonstrating the feasibility of using mixed ionic guest units to drive symmetry reduction in crystal structures.

**Table 1:** Wyckoff sites of Ba[Ba_6_Ch][Si_12_P_20_], Sr[Sr_6_Ch][Si_12_P_20_] and Ba[Ba_2_Sr_4_Ch][Si_12_P_20_]

Ba[Ba_6_Ch][Si_12_P_20_]	Ba1	Ba2	Si1	P1	P2	Ch1
	24e	4b	48g	48i	32f	4a
Sr[Sr_6_Ch][Si_12_P_20_]	Sr1	Sr2	Si1	P1	P2	Ch1
	24e	4b	48g	48i	32f	4a
Ba[Ba_2_Sr_4_Ch][Si_12_P_20_]	Ba1/Sr1	Ba2	Si1, Si2	P1	P2, P3	Ch1
	24f	4b	24g	48h	16e	4a

**Fig. 2:**
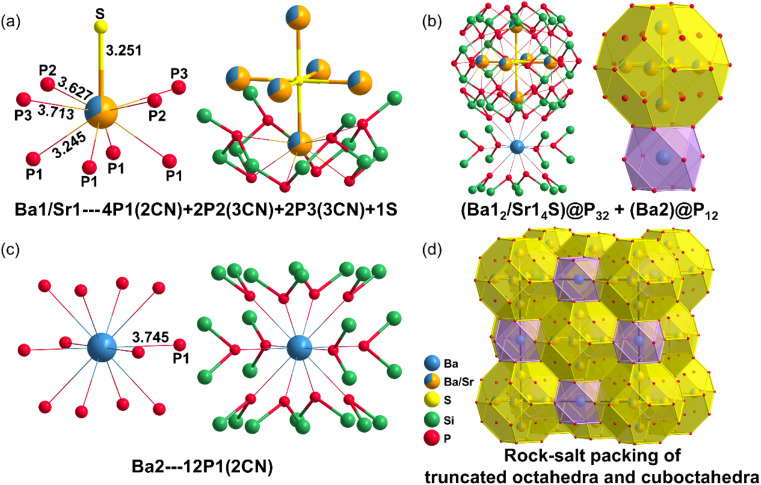
The coordination environments of Ba1 and Ba2 atoms in Ba[Ba_2_Sr_4_S][Si_12_P_20_]. (a) The coordination environment of Ba1. (b) The coordination environment of Ba2. (c) The truncated octahedron (Ba1_2_/Sr1_4_S)@P_32_ and cuboctahedron (Ba2)@P_12_. (d) The rock-salt sublattice packing in Ba[Ba_2_Sr_4_S][Si_12_P_20_].

The coordination environments of Ba1/Sr1 and Ba2 in the ionic units differ significantly in Ba[Ba_2_Sr_4_S][Si_12_P_20_]. The Ba1/Sr1 atom in [Ba_2_Sr_4_S] interacts with four 2CN P1 atoms, two 3CN P2 atoms, two 3CN P3 atoms, and one S atom (Fig. 2a). The Ba1/Sr1–S (3.251 Å) and Ba1/Sr1–P1 (3.245 Å) bonds exhibit some covalent character, while the Ba1/Sr1–P2 (3.627 Å) and Ba1/Sr1–P3 (3.713 Å) bonds are less covalent. The isolated Ba2 atom interacts with twelve 2CN P1 atoms (Fig. 2b), with Ba2–P1 (3.745 Å) showing weak covalent character. The [Ba_2_Sr_4_S] unit coordinates with thirty-two surrounding P atoms to form the truncated octahedron (Ba1_2_/Sr1_4_S)@P_32_, with twenty-four P1 atoms at the vertices and four P1 and four P2 atoms located at the centers of the hexagonal faces of the truncated octahedron (Fig. 2c). The isolated Ba2 atom coordinates with twelve 2CN P1 atoms to form the cuboctahedron (Ba2)@P_12_. The large, truncated octahedron (Ba1_2_/Sr1_4_S)@P_32_ and the cuboctahedron (Ba2)@P_12_ share square faces and are packed together in a rock-salt sublattice arrangement (Fig. 2d). The distance between two diagonally opposed P atoms in the square face shared by the cuboctahedron and the truncated octahedron is 5.296 Å. The channel size between Ba1/Sr1 and Ba2 is comparable to that of zeolites, typically ranging from 4.2 Å to 7.4 Å. Ba[Ba_6_S][Si_12_P_20_] exhibits a similar coordination environment (Fig. S1a–d).

The powder XRD patterns of the target compounds show excellent fitting results, confirming the reliability of the single-crystal structure determination (Fig. S2a–h). Energy-dispersive spectroscopy (EDS) indicates that the elemental ratio of Ba/Si/P/S in Ba[Ba_6_S][Si_12_P_20_] is 7.06/11.99/19.93/1.02, consistent with the single-crystal XRD result of 7/12/20/1. Meanwhile, the elemental ratio of Ba/Sr/Si/P/S in Ba[Ba_2_Sr_4_S][Si_12_P_20_] is 3.02/3.95/12.01/20.06/0.96, consistent with the single-crystal XRD result of 3/4/12/20/1. The remaining compounds in this series all exhibit similar consistency in elemental ratios (Fig. S3a–h).

**Fig. 3:**
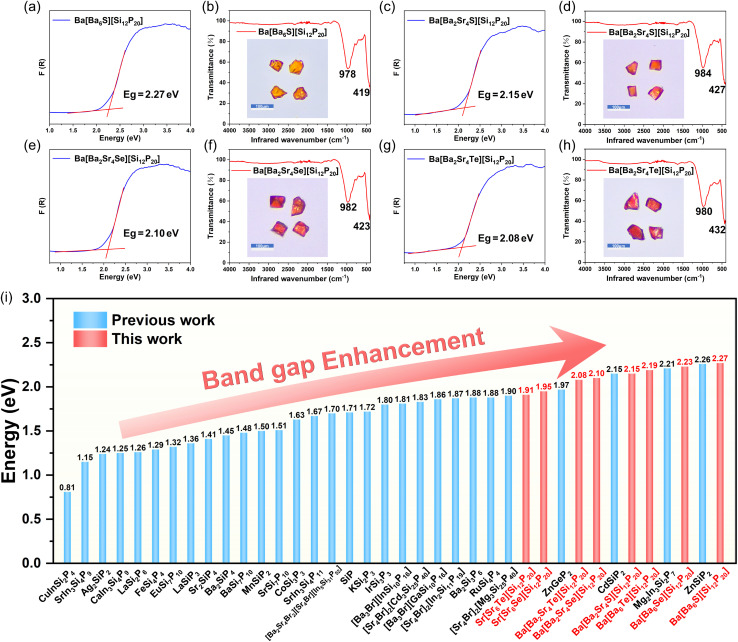
Optical properties. (a, c, e and g) UV-vis-NIR diffuse reflectance spectra and band gap values. (b, d, f and h) IR ATR transmission spectra with the corresponding crystal morphologies shown in insets. (i) A bar chart comparing the band gaps of A–M–Pn–X and A–Si–P silicophosphides.

Diffuse reflectance measurements reveal that the powder band gap values of this series of chalcogenide-inclusion pnictides are 2.27, 2.23, 2.19, 2.15, 2.10, 2.08, 1.95, and 1.91 eV (Fig. 3a, c, e, g, S4a, c, e, and g). The band gap sizes correlate with the variation in crystal color. To our knowledge, these values represent the highest reported band gaps for pnictides in the Ba/Sr–Si–P system.^30–43^ This indicates that even a very small proportion of chalcogen atoms (Ch/P = 1/20) plays a crucial role in enhancing the band gap. Furthermore, these compounds exhibit significant band gap advantages compared to other silicophosphides such as A–M–Pn–X and A–Si–P (Fig. 3i).

Raman spectroscopy was used to analyze the vibrational modes of the chalcogenide-including pnictides: low-frequency peaks primarily originate from Ba/Sr–S/Se/Te bond vibrations, mid-frequency peaks arise from Ba–P and Si–P bond vibrations, and high-frequency peaks are attributed to Si–P bond vibrations (Fig. S5). IR ATR transmission spectra show no significant absorption peaks in these crystals up to approximately 10.2 µm (∼980 cm^−1^) (Fig. 3b, d, f, h, S4b, d, f and h). Their IR transmission cutoff ranges are comparable to those of other silicophosphides like CdSiP_2_ (∼10.0 µm),^40^ Ag_2_SiP_2_ (∼10.0 µm),^11^ MgSiP_2_ (∼10.3 µm)^12^ and [Sr_4_Br]_2_[Mg_3_Si_25_P_40_] (∼9.4 µm).^25^ This is because their IR absorption edges are determined by the two-phonon absorption of Si–P bond vibrations (*ν*: ∼1010 cm^−1^). Thermogravimetric (TG) analysis indicates that Ba[Ba_6_Ch][Si_12_P_20_] remains stable without decomposition within the temperature range of 40–900 °C (Fig. S6). The thermal stability surpasses those of most salt-inclusion pnictides and chalcogenides, attributable to the synergistic effect of the rigid Si–P covalent framework and the polar ionic-covalent Ba–S/Se/Te bonds.

**Fig. 4:**
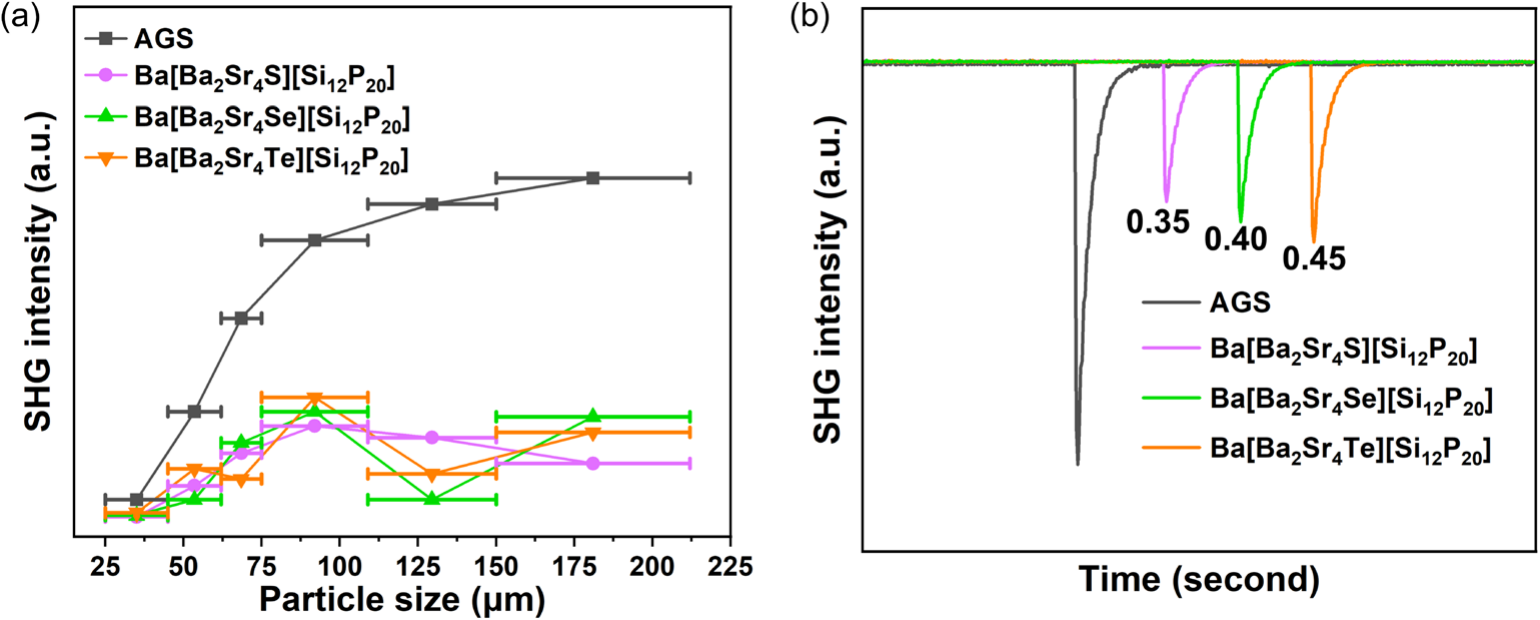
Nonlinear optical properties. (a) Plots of SHG signal *versus* particle size. (b) Measured SHG signals at a particle size of 75–106 µm.

The powder SHG responses were evaluated using the Kurtz-Perry method with AgGaS_2_ (AGS) as a reference standard.^44^ As Ba[Ba_2_Sr_4_Ch][Si_12_P_20_] crystallizes in a cubic system, it is expected to exhibit non-type-I phase-matching behavior (Fig. 4a). Under laser irradiation at a wavelength of 2.05 µm, polycrystalline samples of Ba[Ba_2_Sr_4_S][Si_12_P_20_], Ba[Ba_2_Sr_4_Se][Si_12_P_20_], and Ba[Ba_2_Sr_4_Te][Si_12_P_20_] with particle sizes of 75–106 µm exhibited SHG intensities 0.35, 0.40, and 0.45 times that of AGS, respectively (Fig. 4b). These results indicate that the [Ba_2_Sr_4_Ch] mixed-occupancy octahedral ionic units effectively break the inversion symmetry of the structure, despite the overall high symmetry of Ba[Ba_2_Sr_4_Ch][Si_12_P_20_]. Furthermore, several other non-centrosymmetric cubic compounds have also been reported to display SHG effects with NPM behavior, such as Zn_3_PI_3_ (2.7× AGS, *F4̄*3*m*),^45^ Cu_6_PS_5_Br (2.0× AGS, *F4̄*3*m*),^46^ Cu_10_Te_4_S_13_ (3.75× AGS, *I4̄*3*m*),^47^ Na_4_[Al_3_(BO_3_)_4_]·Cl (1.1× KDP, *P4̄*3*n*),^28^ Na_2_Ba_7_Sn_4_Se_16_ (0.2× AGS, *I4̄*3*d*)^48^ and Pb_4_Ti_3_TeO_13_ (0.4× AGS, *F4̄*3*m*).^49^ To further confirm the SHG signal, we measured the SHG intensity as a function of incident laser power (6.2–14.3 mW) for Ba[Ba_2_Sr_4_Ch][Si_12_P_20_] (Ch = S, Se, Te).^50,51^ In linear coordinates, the goodness of fit (*R*^2^) values are 0.998, 0.997, and 0.999 (Fig. S7a, c and e). In double-logarithmic coordinates, the SHG intensities exhibit a good linear relationship with the incident laser power, with fitted slopes of 1.98, 1.94, and 1.97 (Fig. S7b, d and f). These values agree with the quadratic dependence *I*_SHG_ ∝ (*P*_*ω*_)^2^, confirming that the observed signals indeed originate from the second-harmonic generation effect.

**Fig. 5:**
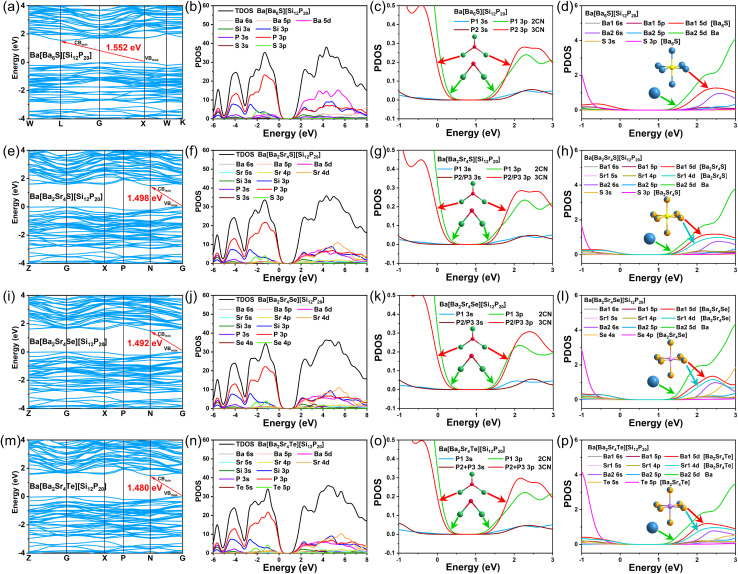
Band structures and PDOS. (a, e, i and m) Band structure diagrams, (b, f, j and n) density of states (DOS) diagrams, (c, g, k and o) projected density of states (PDOS) diagrams of P1 (2CN) and P2/P3 (3CN) atoms, and (d, h, l and p) PDOS diagrams of Ba, [Ba_6_S], [Ba_2_Sr_4_S], [Ba_2_Sr_4_Se], and [Ba_2_Sr_4_Te] for Ba[Ba_6_S][Si_12_P_20_], Ba[Ba_2_Sr_4_S][Si_12_P_20_], Ba[Ba_2_Sr_4_Se][Si_12_P_20_] and Ba[Ba_2_Sr_4_Te][Si_12_P_20_].

To gain deeper insight into the regulatory mechanism of ionic units on the band gap of chalcogenide-including pnictides, we conducted systematic first-principles calculations. Band structure calculations reveal that Ba[Ba_6_Ch][Si_12_P_20_] (Ch = S, Se, Te) and Ba[Ba_2_Sr_4_Ch][Si_12_P_20_] (Ch = S, Se, Te) exhibit indirect band gaps with calculated values of 1.552 eV, 1.547 eV, 1.533 eV, 1.498 eV, 1.492 eV, and 1.480 eV, respectively (Fig. 5a, e, i, m, S8a, e, i and m). When Ba in the guest units is fully replaced by Sr, the band gaps of Sr[Sr_6_Ch][Si_12_P_20_] (Ch = Se, Te) further narrow and transition to direct band gaps of 1.285 eV and 1.252 eV, respectively. Although the generalized gradient approximation (GGA) functional generally underestimates absolute band gap values, the trend in band gap variation is fully consistent with the experimental results, validating the reliability of the calculations. Density of states (DOS) analysis elucidates the contributions of atomic orbitals to the band structure. The chalcogenide-including pnictides exhibit similar distributions near the Fermi level, with the valence band maximum (VBM) dominated by P-3p orbitals and the conduction band minimum (CBM) primarily composed of Ba-5d/Sr-4d, Si-3p, and P-3p orbitals (Fig. 5b, f, j, n, S8b, f, j, and n). The Ba/Sr orbitals tend to occupy positions closer to the Fermi level in the conduction band, indicating that the optical band gap is mainly governed by Ba/Sr–P interactions.

A comparison of P atoms in different coordination environments reveals that the covalent coordination number of P significantly influences the band gap. The 3p orbitals of 3CN P2 atoms are flatter than those of 2CN P1 atoms, contributing more significantly to the band gap (Fig. 5c, g, k, o, S8c, g, k, and o). This validates our previous viewpoint that higher P coordination numbers (CN = 3/4) favor band gap widening. Notably, the chalcogenide-including pnictides in this work share similar P covalent coordination environments with previously reported Ba/Sr–Si–P systems and A–M–Pn–X type compounds: P atoms are primarily 2CN and 3CN, with only [Sr_4_Br][In_2_Si_11_P_19_] containing 4CN P atoms with non-bonding electrons, which are more favorable for band gap broadening. Conventionally, having a higher proportion of 4CN and 3CN P atoms in pnictides is believed to benefit band gap widening. However, in this work, despite an average P coordination number (ACN) of only 2.4, the band gaps reach 1.91–2.27 eV (Table S7). In contrast, many compounds with higher ACN values exhibit narrower band gaps, such as Ba_2_Si_3_P_6_ (ACN = 2.42; 1.88 eV), BaSi_7_P_10_ (ACN = 2.80; 1.48 eV), [Sr_4_Br]_2_[Mg_3_Si_25_P_40_] (ACN = 2.80; 1.90 eV), [Ba_3_Br][GaSi_10_P_16_] (ACN = 2.75; 1.86 eV), [Sr_4_Br][In_2_Si_11_P_19_] (ACN = 2.68; 1.87 eV) and [Ba_2_Sr_4_Br_2_][Sr_4_Br][In_5_Si_31_P_52_] (ACN = 2.77; 1.70 eV). This suggests that besides the positive contribution of 3CN P in the covalent framework, the ionic units [Ba_6_Ch] and isolated Ba2 likely play a critical role in band gap widening.

To verify this hypothesis, we further analyzed the electronic effects of chalcogen elements (Fig. 5d, h, l, p, S8d, h, l and p). The S-3p orbitals reside in the deep valence band (−3 eV to −1 eV) at the lowest energy levels, effectively lowering the VBM and contributing most significantly to band gap broadening. As the atomic number of the chalcogen increases, the Se-4p orbitals are distributed between −2.5 eV and −0.5 eV, while the Te-5p orbitals lie between −2.0 eV and 0 eV, with sequentially higher orbital energy levels. Consequently, their contribution to band gap enhancement diminishes accordingly, aligning with the experimentally observed decreasing trend in band gap values. The DOS plots of Ba1 and Ba2 atoms show that the 5d orbitals of Ba1 in [Ba_6_S] are flatter than those of isolated Ba2. The Ba1–S interaction in [Ba_6_S] effectively suppresses the contribution of Ba1-5d to the CBM. Combined with the shorter Ba1–P bond lengths, the Ba1 sites in the [Ba_6_S] ionic unit enhance localized covalent interactions, making them more favorable for band gap widening compared to the isolated Ba2 sites. Similarly, in [Ba_2_Sr_4_Ch], Ba1 and Sr1 exhibit flatter orbitals relative to isolated Ba2, and in [Sr_6_Ch], Sr1 shows flatter orbitals relative to isolated Sr2. In Ba[Ba_6_Ch][Si_12_P_20_] and Ba[Ba_2_Sr_4_Ch][Si_12_P_20_], the detrimental effect of the empty orbitals at isolated Ba2 on the conduction band minimum primarily originates from the 5d orbitals, while the contribution of the 6s orbitals is negligible due to their excessively low energy levels and highly diffuse nature. In contrast, in Sr[Sr_6_Ch][Si_12_P_20_], the 5s orbitals at isolated Sr2 sites exhibit an even stronger band-gap-narrowing effect than the 4d orbitals. This is attributed to the fact that, compared to the 6s orbitals of Ba2, the 5s orbitals of Sr2 have a smaller principal quantum number, higher energy levels, and a more spatially compact distribution. These characteristics lead to significantly enhanced energy matching and spatial overlap with the P-3p orbitals of the [Si_12_P_20_] framework, resulting in a more pronounced band-gap-narrowing effect.

**Fig. 6:**
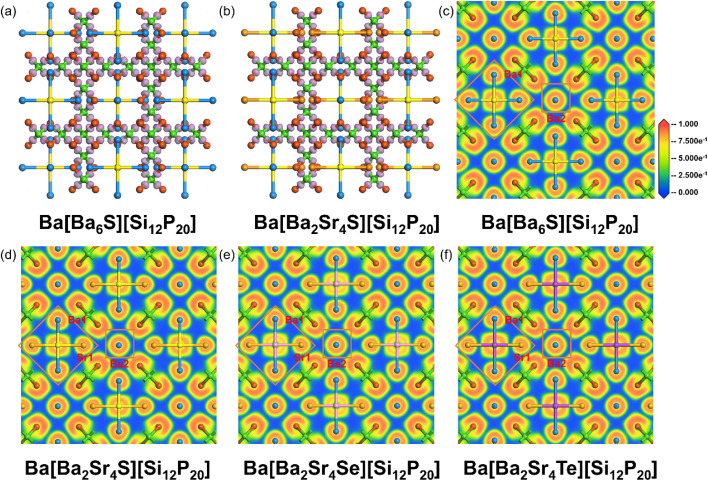
EDD and ELF diagrams. (a and b) EDD isosurface distributions of Ba[Ba_6_S][Si_12_P_20_] and Ba[Ba_2_Sr_4_S][Si_12_P_20_], and (c, d, e and f) slices of ELF field distributions of Ba[Ba_6_S][Si_12_P_20_], Ba[Ba_2_Sr_4_S][Si_12_P_20_], Ba[Ba_2_Sr_4_Se][Si_12_P_20_] and Ba[Ba_2_Sr_4_Te][Si_12_P_20_].

Electron density difference (EDD) analysis reveals significant electron accumulation at the midpoint of each Si–P bond within the SiP_4_ tetrahedra, confirming the strong covalent character of the Si–P bonds (Fig. 6a and b). Electron localization function (ELF) analysis provides an intuitive electronic structure perspective for understanding the band gap broadening mechanism (Fig. 6c–f and S9a–d). In the [Ba_6_S] unit, the S atom is surrounded by highly localized square-shaped electron density, characteristic of typical six-coordinated ionic bonding. The Ba1 atom coordinated to S exhibits noticeable electron polarization, indicating non-negligible covalent character in the Ba1–S bond. This covalency enables the S atom to effectively modulate charge transfer from Ba1 to the framework P atoms, establishing a weak directional interaction between Ba1 and P. This promotes electron localization, suppresses the “electron leakage” effect of P anions, and ultimately reduces the metallicity of the system, while significantly widening the band gap. In contrast, the A–X bonds (*e.g.*, Ba–Br) in salt-including pnictides are highly ionic; although they can increase the band gap, they often simultaneously enhance the metallic character of A–P interactions. The A–Ch bonds (*e.g.*, Ba–S) exhibit stronger covalency, leading to more localized charge distribution throughout the structure. The isolated Ba2 atom displays a spherical electron density distribution, consistent with metallic Ba sites in the known compounds BaSi_7_P_10_ and BaGe_2_P_2_ (Fig. S10a–d), confirming the “polar metallic” nature of the Ba2–P bonds, which do not contribute to band gap widening; this is consistent with observations from PDOS analysis.

Furthermore, the chemical bonding nature within the [Ba_2_Sr_4_Ch] units evolves systematically with the central anion. The highly localized electron density around the S atom indicates strong ionic interactions with limited covalent regions. The more diffuse 4p orbitals of the Se atom exhibit significant overlap with Ba/Sr orbitals, forming a spatially extended and more delocalized covalent bonding network. The larger atomic radius and more diffuse 5p orbitals of the Te atom result in the reappearance of highly localized electron density regions with greater spatial extension, showing stronger covalent character. The evolution in bonding mode and electron localization degree—from S (localized ionic-covalent bonding) to Se (delocalized ionic-covalent bonding) to Te (localized covalent bonding)—closely aligns with the experimentally observed decreasing trend in band gap. Multiple dimensions of evidence—including band structures, density of states distributions, chemical bonding characteristics, and electron localization behavior—collectively confirm that the [Ba_6_Ch]/[Ba_2_Sr_4_Ch]/[Sr_6_Ch] ionic units synergistically achieve significant band gap widening in chalcogenide-including pnictides of the type A[A_6_Ch][Si_12_P_20_] (A = Sr, Ba; Ch = S, Se, Te) through localized covalent interactions, optimized charge transfer pathways, and the effective suppression of system metallicity.

## Conclusions

We designed and synthesized the first series of A–M–Pn–Ch-phase pnictides A[A_6_Ch][Si_12_P_20_] (A = Sr, Ba; Ch = S, Se, Te) with interpenetrated topology structures. They exhibit wide band gaps of 1.91–2.27 eV, significantly outperforming known pnictides from the Ba/Sr–Si–P and A–M–Pn–X systems. The formation of wide band gaps primarily originates from electronic-structure modulation by the [A_6_Ch] octahedral ionic units, whose moderate ionic-covalent hybrid bonding characteristics effectively promote charge localization within the system while suppressing metallic behavior. Notably, even at an extremely low Ch/P ratio of 1/20, the [A_6_Ch] octahedral ionic units still significantly widen the band gap. The new A–M–Pn–Ch phase of pnictides established in this work provides a previously unreported feasible pathway for designing wide-band-gap pnictides.

## Author contributions

Huikang Jiang: investigation, data curation, formal analysis, writing – original draft. Guang Peng: conceptualization, methodology, validation. Ning Ye: supervision, writing – review & editing. Jindong Chen: supervision, conceptualization, writing – review & editing.

## Conflicts of interest

There are no conflicts to declare.

## Supplementary Material

SC-017-D6SC00313C-s001

SC-017-D6SC00313C-s002

## Data Availability

CCDC 2522057–2522064 contain the supplementary crystallographic data for this paper.^52*a*–*h*^ All supplementary data relating to the results of this study are available in the article and its supplementary information (SI) file. Supplementary information is available. See DOI: https://doi.org/10.1039/d6sc00313c.
