# Supplementary material for: A[A6Ch][Si12P20] (A = Sr, Ba; Ch = S, Se, Te): achieving a wide band gap in pnictides by constructing [A6Ch] octahedral ionic units
Source: Chem Sci. 2026 May 13;17(25):12541–50. doi: 10.1039/d6sc00313c (PMC13183291; doi:10.1039/d6sc00313c)
Supplement: SC-017-D6SC00313C-s001 [file SC-017-D6SC00313C-s001.pdf]

Supplementary Information (SI) for Chemical Science.
This journal is © The Royal Society of Chemistry 2026

Supporting Information

$A[A_6Ch][Si_{12}P_{20}]$ (A = Sr, Ba; Ch = S, Se, Te): Achieving Wide Band Gap of Pnictides by Constructing $[A_6Ch]$ octahedral ionic units

Huikang Jiang[1,3], Guang Peng,[1] Ning Ye[1,2] *, Jindong Chen[1,2] *

[1]State Key Laboratory of Crystal Materials, Tianjin Key Laboratory of Functional Crystal Materials, Institute of Functional Crystal, College of Materials Science and Engineering, Tianjin University of Technology, Tianjin 300384 (China)
[2]Tianjin Key Laboratory of Quantum Optics and Intelligent Photonics, School of Science, Tianjin University of Technology, Tianjin 300384 (China)
[3]SEU-FEI Nano-Pico Center, Key Laboratory of MEMS of Ministry of Education, School of Integrated Circuit, Southeast University, Nanjing 210096 (China)
*E-mail: nye@email.tjut.edu.cn, cjd1225@email.tjut.edu.cn

Table of Contents

**Section**	**Title**	**Page**
	Experimental Section	2
Table S1	Crystal data and structure refinement for $Ba[Ba_6S][Si_{12}P_{20}]$, $Ba[Ba_6Se][Si_{12}P_{20}]$, $Ba[Ba_6Te][Si_{12}P_{20}]$, $Ba[Ba_2Sr_4S][Si_{12}P_{20}]$, $Ba[Ba_2Sr_4Se][Si_{12}P_{20}]$, $Ba[Ba_2Sr_4Te][Si_{12}P_{20}]$, $Sr[Sr_6Se][Si_{12}P_{20}]$ and $Sr[Sr_6Te][Si_{12}P_{20}]$.	5
Table S2	Atomic Coordinates ($\times10^4$) and Equivalent Isotropic Displacement Parameters ($Å^2\times10^3$).	8
Table S3	Selected bond lengths (Å).	10
Table S4	Selected bond angles (degree).	11
Table S5	Anisotropic Displacement Parameters ($Å^2\times10^3$).	13
Table S6	Crystallographic information of compounds containing six-coordinated $[A_6Q]/[A_6X]$ ionic group.	16
Table S7	Average coordination number (ACN) of Ba/Sr-Si-P and A-M-Pn-X series compounds.	18
Figure S1	**Coordination environments of Ba1 and Ba2 atoms in $Ba[Ba_6S][Si_{12}P_{20}]$**. (a) Coordination environment of Ba1. (b) Coordination environment of Ba2. (c) Truncated octahedron $(Ba1_6S)@P_{32}$ and cuboctahedron $(Ba2)@P_{12}$. (d) Rock-salt sublattice packing in $Ba[Ba_6S][Si_{12}P_{20}]$.	19
Figure S2	**Powder XRD patterns.** Powder XRD patterns of the experimental and simulated for $Ba[Ba_6S][Si_{12}P_{20}]$ (a), $Ba[Ba_6Se][Si_{12}P_{20}]$ (b), $Ba[Ba_6Te][Si_{12}P_{20}]$ (c), $Ba[Ba_2Sr_4S][Si_{12}P_{20}]$ (d), $Ba[Ba_2Sr_4Se][Si_{12}P_{20}]$ (e), $Ba[Ba_2Sr_4Te][Si_{12}P_{20}]$ (f), $Sr[Sr_6Se][Si_{12}P_{20}]$ (g) and $Sr[Sr_6Te][Si_{12}P_{20}]$ (h).	20
Figure S3	**Energy-dispersive X-ray spectroscopy (EDS) analysis.** EDS spectra of $Ba[Ba_6S][Si_{12}P_{20}]$ (a), $Ba[Ba_6Se][Si_{12}P_{20}]$ (b), $Ba[Ba_6Te][Si_{12}P_{20}]$ (c), $Ba[Ba_2Sr_4S][Si_{12}P_{20}]$ (d), $Ba[Ba_2Sr_4Se][Si_{12}P_{20}]$ (e), $Ba[Ba_2Sr_4Te][Si_{12}P_{20}]$ (f), $Sr[Sr_6Se][Si_{12}P_{20}]$ (g) and $Sr[Sr_6Te][Si_{12}P_{20}]$ (h).	21
Figure S4	**Optical properties.** (a, c, e and g) UV-Vis-NIR diffuse reflectance spectra and band gap values. (b, d, f and h) IR ATR transmission spectra and corresponding crystal crystal photos (inset b，d，f and h).	22
Figure S5	**Raman scattering spectra.** Raman scattering spectra of $Ba[Ba_6S][Si_{12}P_{20}]$, $Ba[Ba_6Se][Si_{12}P_{20}]$, $Ba[Ba_6Te][Si_{12}P_{20}]$, $Ba[Ba_2Sr_4S][Si_{12}P_{20}]$, $Ba[Ba_2Sr_4Se][Si_{12}P_{20}]$, $Ba[Ba_2Sr_4Te][Si_{12}P_{20}]$, $Sr[Sr_6Se][Si_{12}P_{20}]$ and $Sr[Sr_6Te][Si_{12}P_{20}]$.	22
Figure S6	**TG curves**. TG curves of $Ba[Ba_6S][Si_{12}P_{20}]$, $Ba[Ba_6Se][Si_{12}P_{20}]$ and $Ba[Ba_6Te][Si_{12}P_{20}]$.	23
Figure S7	**Power-dependent SHG measurements** (a, c, e) Linear-scale plots of SHG intensity as a function of incident laser power for $Ba[Ba_2Sr_4S][Si_{12}P_{20}]$, $Ba[Ba_2Sr_4Se][Si_{12}P_{20}]$, and $Ba[Ba_2Sr_4Te][Si_{12}P_{20}]$, respectively; (b, d, f) the corresponding double-logarithmic plots.	23
Figure S8	**Band structures and PDOS**. (a,e,i,m) Band structure diagrams, (b,f,j,n) Density of states (DOS) diagrams, (c,g,k,o) Projected density of states (PDOS) of P1 (2CN), P2/P3 (3CN) atoms. (d,h,l,p) PDOS of Ba and $[Ba_6Se]$, $Ba[Ba_6Te]$, $[Sr_6Se]$, $[Sr_6Te]$.	24
Figure S9	**ELF diagrams**. Slice electron localization function (ELF) field distribution of of $Ba[Ba_6Se][Si_{12}P_{20}]$ (a), $Ba[Ba_6Te][Si_{12}P_{20}]$ (b), $Sr[Sr_6Se][Si_{12}P_{20}]$ (c) and $Sr[Sr_6Te][Si_{12}P_{20}]$ (d).	25
Figure S10	**Crystal structure and ELF diagrams**. (a, b) Crystal structures of $BaSi_7P_{10}$ and $BaGe_2P_2$; (c, d) corresponding slice ELF field distributions.	26

Experimental Section

Raw Materials.

The raw materials were Ba (5N, Adamas), Sr (4N, Adamas), Si (5N, Adamas), P (5N, Adamas), S (4N, Adamas), Se (4N, Adamas), Te (4N, Adamas), KI (5N, Adamas) and $SrBr_2$ (5N, Adamas), which were not further purified.

Salt-Flux Synthesis.

$Ba[Ba_6S][Si_{12}P_{20}]$, $Ba[Ba_6Se][Si_{12}P_{20}]$, $Ba[Ba_6Te][Si_{12}P_{20}]$, $Sr[Sr_6Se][Si_{12}P_{20}]$ and $Sr[Sr_6Te][Si_{12}P_{20}]$: Ba/Sr, Si, P, S/Se/Te and KI at a molar ratio of 7:12:20:1:12-20 are mixed and thoroughly ground, and then placed in quartz tubes which is evacuated to 1×10^{-3} Pa and flame-sealed. The tubes were placed in a muffle furnace and was heated to 900°C/900°C/950°C/800°C/850°C, hold at that temperature for 96-120 hours, slowly cooled at -5°C/h to 500°C, and then cool it to room temperature at -10°C/h. The quartz tube is opened in a well-ventilated area, and the product is washed using deionized water to remove the KI flux. The yield of title compounds was up to 95%

$Ba[Ba_2Sr_4S][Si_{12}P_{20}]$, $Ba[Ba_2Sr_4Se][Si_{12}P_{20}]$, $Ba[Ba_2Sr_4Te][Si_{12}P_{20}]$: Ba, Si, P, S/Se/Te and $SrBr_2$ at a molar ratio of 7:12:20:1: 20 are mixed. Other operations were similar to the former, except that the constant temperature should be 1000°C/1000°C/1050°C, respectively.

Single-Crystal X-Ray Diffraction.

The diffraction data were collected on a Bruker D8 VENTURE diffractometer and Mo Kα radiation (λ = 0.71073 Å). The data was integrated by SHELXL-2018/3, and the multi-scan method was used to the absorption corrections. The crystal structure of the two compounds was determined by the intrinsic phasing methods and refined with anisotropic thermal parameters for all atoms by full-matrix leasts-quares fitting on F2 using SHELXL on Olex2 program.[1,2] The PLATON program was used to check the correctness of the structures, and no higher symmetries were found.[3] The crystal data and structure refinement parameters were shown in Table S1. Some structural parameters including Atomic Coordinates ($\times10^4$) and Equivalent Isotropic Displacement Parameters ($Å^2\times10^3$), Selected bond lengths (Å) and Anisotropic Displacement Parameters ($Å^2\times10^3$) are listed in Table S2, Table S3, and Table S4.

Powder X-Ray Diffraction (PXRD).

The PXRD measurements of compounds were conducted utilizing a Rigaku Smart Lab 9kW diffractometer equipped with a diffracted monochromator setting for Cu K_α radiation (λ=1.5418 Å). The experimental data were characterized at ambient temperature within the 2θ range of 10−70°, at a step size of 0.01° and a step time of 2 s. The powder XRD patterns was in good agreement with the calculated derived from the crystallographic information file

(CIF), which indicates that the pure phase is obtained.

Elemental Analysis.
The elemental composition analysis was performed on a field-emission scanning electron microscope (Quanta FEG 250) equipped with an energy-dispersive X-ray spectrometer.

UV−Vis−NIR transmittance and diffuse reflectance spectrum.
The powder sample was uniformly pressed into a pellet to ensure a flat surface with $BaSO_4$ as 100% reflectance standard. The UV-Vis-NIR diffuse reflectance spectrum was collected using a HITACHI UH4150 spectrophotometer in the range of 190–2500 nm with a scan speed of 300 nm/min. The obtained diffuse reflectance data were processed using the Kubelka-Munk equation $F(R) = \frac{K}{S} = \frac{(1-R)^2}{2R}$ (where R is the diffuse reflectance coefficient, K is the absorption coefficient, and S is the scattering coefficient) The band gap energy was determined by plotting the photon energy $h\nu$ on the x-axis and $F(R)$ on the y-axis, drawing a tangent line at the absorption edge, and extrapolating it to the x-axis. This method provides an important basis for evaluating the electronic structure and optical absorption characteristics of nonlinear optical materials.[4]

IR transmittance spectrum.
The IR spectrum was recorded using a Nicolet iS50 FT-IR spectrometer at room temperature in the range of 400-4000 cm^{-1} for sample. A sample of ~10 mg was used for testing.

Raman Spectroscopy.
The Raman spectra of the compounds were obtained by a confocal microscope laser Raman spectrometer (inVia Qontor) equipped with a CCD detector. The radiation wavelength was 532 nm, and the laser intensity was 5%.

Thermal Analysis.
Thermogravimetric (TG) were performed on a NETZSCH 209F3A unit in N2 atmosphere at 10 °C/min heating rate from 40 to 900°C. Approximate 5-10mg of title compounds were ground into fine powders and enclosed in Al_2O_3 crucibles.

Polycrystalline Second Harmonic Generation (SHG) Measurements.
Polycrystalline SHG responses were measured with the Kurtz-Perry method[5] using Q-switched Nd: YAG solid-state laser of wavelength 2050 nm. For polycrystalline samples were ground and sieved into several distinct particle size ranges of 25-45, 45-58, 58-75, 75-106, 106-150 and 150-212 μm, and then pressed into the container with a thickness of 1mm and diameter of 8 mm.

Polycrystalline $AgGaS_2$ was prepared with the same size range as the comparison reference. Power-dependent SHG responses were measured using an OPO laser system (10 ns, 5 Hz) of wavelength ~3 μm. The incident laser power was tuned from 6.2 to 14.3 mW.

Theoretical Calculation Details.

The electronic structure calculations were performed by the first-principles calculations in the CASTEP package based on density functional theory, with the Norm-conserving pseudopotentials.[6-12] The Perdew-Burke-Ernzerhof (PBE) functional within the generalized gradient approximation (GGA) was applied for the exchange-correlation potential.[13,14] The following orbital electrons were treated as valence electrons, Ba: $4d^{10}5p^{6}6s^{2}$, Sr: $3d^{10}4p^{6}5s^{2}$, Si: $3s^{2}3p^{2}$, P: $3s^{2}3p^{3}$, S: $3s^{2}3p^{4}$/Se: $4s^{2}4p^{4}$/Te: $5s^{2}5p^{4}$. To achieve energy convergence, the plane-wave energy cutoff was set at 720 eV. The Monkhorst-Pack grid size for self-consistent field calculation is 2 × 2 × 2.[15-18] Because of the discontinuity of exchange correlation, bandgaps calculated by the GGA method are usually smaller than experimental values, so a scissor operator was adopted to raise the CBs to match the experimental value for optical property calculation. ELF isosurface labels：The isosurface values range from 0 to 1, with a contour interval of 2.5×10^{-1}. EDD electron cloud settings: The density threshold (Isovalue) for the electron cloud display is set to 0.09, and the type is Normal.

Table S1. Crystal data and structure refinement for $Ba[Ba_6S][Si_{12}P_{20}]$, $Ba[Ba_6Se][Si_{12}P_{20}]$, $Ba[Ba_6Te][Si_{12}P_{20}]$, $Ba[Ba_2Sr_4S][Si_{12}P_{20}]$, $Ba[Ba_2Sr_4Se][Si_{12}P_{20}]$, $Ba[Ba_2Sr_4Te][Si_{12}P_{20}]$, $Sr[Sr_6Se][Si_{12}P_{20}]$ and $Sr[Sr_6Te][Si_{12}P_{20}]$.

**Formula**	**$Ba[Ba_6S][Si_{12}P_{20}]$**	**$Ba[Ba_6Se][Si_{12}P_{20}]$**	**$Ba[Ba_6Te][Si_{12}P_{20}]$**
CCDC Number	2522060	2522062	2522061
Formula weight	1949.92	1996.82	2045.46
Temperature (K)	293.00	293.00	293.00
Radiation	Mo Kα (0.71073 Å)	Mo Kα (0.71073 Å)	Mo Kα (0.71073 Å)
Crystal system	cubic	cubic	cubic
Space group	$Fm\bar{3}m$	$Fm\bar{3}m$	$Fm\bar{3}m$
a (Å)	15.701(5)	15.7503(6)	15.826(8)
b(Å)	15.701(5)	15.7503(6)	15.826(8)
c (Å)	15.701(5)	15.7503(6)	15.826(8)
α (deg)	90	90	90
β (deg)	90	90	90
γ (deg)	90	90	90
Volume ($Å^3$)	3871.0	3907.2(4)	3964.1
Z	4	4	4
ρ_{calc} (g/cm^3)	3.346	3.395	3.427
μ (mm^{-1})	8.267	9.068	8.741
F (000)	3504.0	3576.0	3648.0
Reflections collected	8782	14855	9918
Rint	0.0390	0.0780	0.0428
R_1 [I ≥ 2σ (I)]	0.0110	0.0239	0.0140
wR_2 [I ≥ 2σ (I)]	0.0228	0.0471	0.0327
R_1 [all data]	0.0111	0.0248	0.0151
wR_2 [all data]	0.0228	0.0472	0.0331
Goodness-of-fit on F^2	1.191	1.201	1.065

**Formula**	**$Ba[Ba_2Sr_4S][Si_{12}P_{20}]$**	**$Ba[Ba_2Sr_4Se][Si_{12}P_{20}]$**	**$Ba[Ba_2Sr_4Te][Si_{12}P_{20}]$**
CCDC Number	2522057	2522058	2522059
Formula weight	1751.04	1797.94	1846.58
Temperature (K)	293.00	293.00	293.00
Radiation	Mo Kα (0.71073 Å)	Mo Kα (0.71073 Å)	Mo Kα (0.71073 Å)
Crystal system	cubic	cubic	cubic
Space group	$F\bar{4}3m$	$F\bar{4}3m$	$F\bar{4}3m$
a (Å)	15.5666(6)	15.5813(5)	15.6412(4)
b(Å)	15.5666(6)	15.5813(5)	15.6412(4)
c (Å)	15.5666(6)	15.5813(5)	15.6412(4)
α (deg)	90	90	90
β (deg)	90	90	90
γ (deg)	90	90	90
Volume ($Å^3$)	3772.1(5)	3782.8(4)	3826.6(3)
Z	4	4	4
ρ_{calc} (g/cm^3)	3.083	3.157	3.205
μ (mm^{-1})	9.989	10.868	10.540
F (000)	3216.0	3288.0	3360.0
Reflections collected	5057	2800	4115
Rint	0.1263	0.0255	0.0377
R_1 [I ≥ 2σ (I)]	0.0328	0.0171	0.0168
wR_2 [I ≥ 2σ (I)]	0.0705	0.0400	0.0413
R_1 [all data]	0.0387	0.0179	0.0186
wR_2 [all data]	0.0726	0.0402	0.0421
Flack x TWIN/ Parsons	0.46(9)	0.50(8)	0.35(9)
Hooft y	0.08(7)	0.07(4)	0.03(5)
Goodness-of-fit on F^2	1.059	1.182	1.105

**Formula**	**$Sr[Sr_6Se][Si_{12}P_{20}]$**	**$Sr[Sr_6Te][Si_{12}P_{20}]$**
CCDC Number	2522064	2522063
Formula weight	1648.78	1697.420
Temperature (K)	293.00	293.00
Radiation	Mo Kα (0.71073 Å)	Mo Kα (0.71073 Å)
Crystal system	cubic	cubic
Space group	$Fm\bar{3}m$	$Fm\bar{3}m$
a (Å)	15.5303(15)	15.6033(6)
b(Å)	15.5303(15)	15.6033(6)
c (Å)	15.5303(15)	15.6033(6)
α (deg)	90	90
β (deg)	90	90
γ (deg)	90	90
Volume ($Å^3$)	3745.8(11)	3798.8(3)
Z	4	4
ρ_{calc} (g/cm^3)	2.924	2.968
μ (mm^{-1})	12.113	11.738
F (000)	3072.0	3114.6
Reflections collected	9442	9550
Rint	0.0851	0.1064
R_1 [I ≥ 2σ (I)]	0.0309	0.0179
wR_2 [I ≥ 2σ (I)]	0.0734	0.0357
R_1 [all data]	0.0487	0.0261
wR_2 [all data]	0.0794	0.0375
Goodness-of-fit on F^2	1.157	1.035

Table S2. Atomic Coordinates (×10^4) and Equivalent Isotropic Displacement Parameters (Å^2×10^3) and bond valence sums (BVS).

Atom	Wyckoff site	occupancy	x	y	z	U(eq)	BVS
				**$Ba[Ba_6S][Si_{12}P_{20}]$**			
Ba1	24e	1	2064.0(2)	5000	5000	13.96(11)	1.98
Ba2	4b	1	5000	5000	5000	70.8(4)	1.85
Si1	48g	1	2500	2500	4124.2(5)	9.17(17)	3.90
P1	48i	1	3289.8(3)	3289.8(3)	5000	11.17(17)	2.89
P2	32f	1	1643.6(3)	3356.4(3)	3356.4(3)	9.5(2)	3.30
S1	4a	1	0	5000	5000	12.1(5)	1.88
				**$Ba[Ba_6Se][Si_{12}P_{20}]$**			
Ba1	24e	1	5000	5000	2093.2(4)	15.2(2)	2.05
Ba2	4b	1	5000	5000	5000	84.5(12)	1.92
Si1	48g	1	7500	4121.4(13)	2500	10.7(4)	3.86
P1	48i	1	6713.7(8)	5000	3286.3(8)	11.3(4)	2.88
P2	32f	1	6644.5(9)	3355.5(9)	1644.5(9)	10.3(5)	3.25
Se1	4a	1	5000	5000	0	13.2(6)	1.94
				**$Ba[Ba_6Te][Si_{12}P_{20}]$**			
Ba1	24e	1	5000	5000	2147.0(2)	16.26(15)	2.21
Ba2	4b	1	5000	5000	5000	115.1(10)	1.96
Si1	48g	1	7500	4117.6(7)	2500	9.8(2)	3.83
P1	48i	1	6721.4(4)	5000	3278.6(4)	11.6(2)	2.92
P2	32f	1	6648.0(5)	3352.0(5)	1648.0(5)	10.4(3)	3.19
Te1	4a	1	5000	5000	0	16.7(2)	2.14
				**$Ba[Ba_2Sr_4S][Si_{12}P_{20}]$**			
Ba1/Sr1	24f	0.33/0.67	7911.4(7)	5000	5000	22.5(4)	1.89
Ba2	4b	1	5000	5000	5000	63.9(9)	1.82
Si1	24g	1	7500	5868(6)	7500	13(3)	3.83
Si2	24g	1	7500	4114(5)	7500	10(3)	4.12

P1	48h	1	6700.9(10)	4994(5)	6700.9(10)	14.6(6)	2.76
P2	16e	1	8387(5)	6613(5)	6613(5)	7(2)	3.11
P3	16e	1	8341(5)	3341(5)	6659(5)	15(3)	3.57
S1	4a	1	10000	5000	5000	14.4(15)	1.88
**$Ba[Ba_2Sr_4Se][Si_{12}P_{20}]$**							
Ba1/Sr1	24f	0.33/0.67	5000	2109.1(3)	5000	18.1(2)	1.91
Ba2	4b	1	5000	5000	5000	51.5(4)	1.84
Si1	24g	1	2500	2500	4126(5)	7.1(16)	3.89
Si2	24g	1	2500	2500	5879(5)	11.3(17)	4.01
P1	48h	1	3297.8(5)	3297.8(5)	4998(4)	11.7(3)	2.78
P2	16e	1	3378(3)	1622(3)	3378(3)	9.9(17)	3.18
P3	16e	1	3353(3)	1647(3)	6647(3)	9.1(18)	3.40
Se1	4a	1	5000	0	5000	19.2(4)	1.79
**$Ba[Ba_2Sr_4Te][Si_{12}P_{20}]$**							
Ba1/Sr1	24f	0.33/0.67	7841.4(4)	5000	5000	20.0(2)	1.93
Ba2	4b	1	5000	5000	5000	59.7(5)	1.85
Si1	24g	1	7500	7500	4111(4)	11.2(15)	3.91
Si2	24g	1	7500	7500	5875(3)	6.1(13)	3.94
P1	48h	1	6707.9(5)	6707.9(5)	5007(4)	11.0(3)	2.79
P2	16e	1	8356(3)	6644(3)	3356(3)	9.0(14)	3.17
P3	16e	1	8368(3)	6632(3)	6632(3)	8.8(14)	3.35
Te1	4a	1	10000	5000	5000	22.7(3)	1.85
**$Sr[Sr_6Se][Si_{12}P_{20}]$**							
Sr1	24e	1	5000	7857.4(6)	5000	36.7(3)	1.91
Sr2	4b	1	5000	5000	5000	235(5)	1.82
Si1	48g	1	7500	7500	5876.2(9)	25.2(4)	4.09
P1	48i	1	6700.5(7)	6700.5(7)	5000	27.3(4)	2.76
P2	32f	1	6634.7(7)	8365.3(7)	6634.7(7)	25.9(4)	3.31
Se1	4a	1	5000	10000	5000	27.8(5)	1.96
**$Sr[Sr_6Te][Si_{12}P_{20}]$**							

Sr1	24e	1	5000	7813.1(5)	5000	22.2(2)	1.94
Sr2	4b	1	5000	5000	5000	189(4)	1.86
Si1	48g	1	5880.4(8)	7500	7500	12.0(3)	3.96
P1	48i	1	5000	6705.8(5)	6705.8(5)	14.2(3)	2.75
P2	32f	1	6636.1(6)	8363.9(6)	6636.1(6)	12.8(3)	3.25
Te1	4a	1	5000	10000	5000	16.1(3)	1.89

Table S3. Selected bond lengths (Å).

Bond	Length/Å	Bond	Length/Å	Bond	Length/Å
**$Ba[Ba_6S][Si_{12}P_{20}]$**		**$Ba[Ba_6Se][Si_{12}P_{20}]$**		**$Ba[Ba_6Te][Si_{12}P_{20}]$**	
Ba1-S1	3.2407(3)	Ba1-Se1	3.2969(7)	Ba1-Te1	3.3979(4)
P1-Si1	2.2286(8)	P1-Si1	2.2322(19)	P1-Si1	2.2332(10)
P1-Si1#9	2.2286(8)	P1-Si1#9	2.2322(19)	P1-Si1#9	2.2332(10)
P2-Si1	2.2517(5)	P2-Si1	2.2553(14)	P2-Si1	2.2593(7)
P2-Si1#10	2.2517(5)	P2-Si1#6	2.2553(14)	P2-Si1#5	2.2593(7)
P2-Si#15	2.2517(5)	P2-Si1#8	2.2553(14)	P2-Si1#8	2.2593(7)
**$Ba[Ba_2Sr_4S][Si_{12}P_{20}]$**		**$Ba[Ba_2Sr_4Se][Si_{12}P_{20}]$**		**$Ba[Ba_2Sr_4Te][Si_{12}P_{20}]$**	
Ba1/Sr1-S1	3.2512(11)	Ba1/Sr1-Se1	3.2866(5)	Ba1/Sr1-Te1	3.3763(6)
P1-Si1	2.223(8)	P1-Si1	2.222(5)	P1-Si1	2.244(5)
P1-Si2	2.230(8)	P1-Si2	2.230(5)	P1-Si2	2.216(5)
P2-Si1	2.271(6)	P2-Si1	2.258(5)	P2-Si1	2.232(4)
P2-Si1#5	2.271(6)	P2-Si1#17	2.259(5)	P2#6-Si1	2.232(4)
P2-Si1#6	2.271(6)	P2-Si1#18	2.259(5)	P2#9-Si1	2.232(4)
P3-Si2	2.209(6)	P3-Si2	2.229(5)	P3-Si2	2.255(4)
P3#7-Si2	2.209(6)	P3#19-Si2	2.229(5)	P3#9-Si2	2.255(4)
		**$Sr[Sr_6Se][Si_{12}P_{20}]$**		**$Sr[Sr_6Te][Si_{12}P_{20}]$**	
		Sr1-Se1	3.3274(10)	Sr1-Te1	3.4123(7)
		P1-Si1	2.2214(14)	P1-Si1	2.2267(12)
		P1-Si1#11	2.2214(14)	P1-Si1#13	2.2267(12)
		P2-Si1	2.2359(10)	P2-Si1	2.2416(8)

	P2-Si1#6	2.2359(10)	P2-Si1#10	2.2416(8)
	P2-Si#12	2.2359(10)	P2-Si#12	2.2416(8)

Table S4. Selected bond angles (degree).

Bond-	Angles/ degree	Bond	Angles/ degree	Bond	Angles/ degree
**Ba[Ba$_6$S][Si$_{12}$P$_{20}$]**		**Ba[Ba$_6$Se][Si$_{12}$P$_{20}$]**		**Ba[Ba$_6$Te][Si$_{12}$P$_{20}$]**	
Si1^{10}-P1-Si1	76.19(4)	Si1^{16}-P1-Si1	76.62(10)	Si1^{14}-P1-Si1	77.42(5)
Si1-P1-Ba1^{9}	97.36(10)	Si1-P1-Ba1	97.95(2)	Si1-P1-Ba1	99.09(12)
Si1-P2-Si1^{4}	106.43(3)	Si1-P2-Si1^{6}	106.39(8)	Si1-P2-Si1^{5}	106.49(4)
Ba1^{3}-S1-Ba1^{2}	180.0	Ba1-Se1-Ba1^{9}	180.0	Ba1^{1}-Te1-Ba1^{2}	180.0
Ba1^{1}-S1-Ba1	90.0	Ba1-Se1-Ba1^{8}	90.0	Ba1^{1}-Te1-Ba1	90.0
Ba1^{3}-Ba1-Ba1^{1}	60.0	Ba1^{1}-Ba1-Ba1^{2}	60.0	Ba1^{3}-Ba1-Ba1^{2}	60.0
Ba1^{3}-Ba1-Ba1^{2}	90.0	Ba1^{4}-Ba1-Ba1^{2}	90.0	Ba1^{3}-Ba1-Ba1^{4}	90.0
P1-Ba1-P1^{4}	70.16(14)	P1-Ba1-P1^{6}	70.95(3)	P1-Ba1-P1^{7}	72.44(17)
P1^{6}-Ba1-P1	108.73(3)	P1-Ba1-P1^{4}	110.31(7)	P1-Ba1-P1^{6}	113.36(4)
P1-Ba1- S1	125.63(14)	P1-Ba1-Se1	124.85(3)	P1-Ba1-Te1	123.32(18)
S1-Ba1-Ba1^{2}	45.0	Se1-Ba1-Ba1^{1}	45.0	Te1-Ba1-Ba1^{2}	45.0
Ba1-Ba2-Ba1^{16}	180.0	Ba1-Ba2-Ba1^{11}	180.0	Ba1-Ba2-Ba1^{11}	180.0
Ba1-Ba2-Ba1^{4}	90.0	Ba1-Ba2-Ba1^{7}	90.0	Ba1-Ba2-Ba1^{7}	90.0
P1^{10}-Si1-P1	103.80(4)	P1^{16}-Si1-P1	103.37(10)	P1^{14}-Si1-P1	102.58(5)
P1-Si1-P2	109.29(14)	P1-Si1-P2	109.37(3)	P1-Si1-P2	109.60(19)
P2^{14}-Si1-P2	115.26(6)	P2^{17}-Si1-P2	115.32(14)	P2^{15}-Si1-P2	115.14(7)
P1-Si1-Si1^{10}	51.90(2)	P1-Si1-Si1^{16}	51.69(5)	P1-Si1-Si1^{14}	51.29(3)
P2-Si1-Si1^{10}	122.37(3)	P2-Si1-Si1^{16}	122.34(7)	P2-Si1-Si1^{14}	122.43(4)
**Ba[Ba$_2$Sr$_4$S][Si$_{12}$P$_{20}$]**		**Ba[Ba$_2$Sr$_4$Se][Si$_{12}$P$_{20}$]**		**Ba[Ba$_2$Sr$_4$Te][Si$_{12}$P$_{20}$]**	
Si1-P1-Si2	75.61(13)	Si1-P1-Si2	75.68(6)	Si1-P1-Si2	76.41(6)
Si1-P1-Ba1/Sr1	97.46(11)	Si1-P1-Ba1/Sr1	97.98(8)	Si1-P1-Ba1/Sr1	98.77(9)
Si2-P1-Ba1/Sr1	97.62(12)	Si2-P1-Ba1/Sr1	97.90(9)	Si2-P1-Ba1/Sr1	99.14(9)
Ba1/Sr1^{1}-P1-Ba1/Sr1	160.87(9)	Ba1/Sr1-P1-Ba1/Sr1^{10}	159.86(4)	Ba1/Sr1-P1-Ba1/Sr1^{11}	157.14(4)
Ba1/Sr1-P2-Ba1/Sr1^{6}	78.7(2)	Ba1/Sr1-P2- Ba1/Sr1^{4}	78.98(14)	Ba1/Sr1^{2}-P2-Ba1/Sr1^{1}	79.72(14)

Si1-P2-Ba1/Sr1[6]	161.1(5)	Si1-P2-Ba1/Sr11[2]	160.9(3)	Si1-P2-Ba1/Sr1[2]	160.5(3)
Si1-P2-Ba1/Sr1	86.7(2)	Si1-P2-Ba1/Sr11[4]	86.34(15)	Si1-P2-Ba1/Sr1[5]	85.35(14)
Si1[8]-P2-Si1	104.6(5)	Si1-P2-Si1[18]	105.0(3)	Si1-P2-Si1[8]	106.0(3)
Ba1/Sr1[5]-P3-Ba1/Sr1[7]	76.5(2)	Ba1/Sr1[4]-P3- Ba1/Sr1[1]	77.82(12)	Ba1/Sr1[5]-P3- Ba1/Sr1	80.28(14)
Si2-P3-Ba1/Sr1[7]	157.4(5)	Si2-P3-Ba1/Sr1[1]	158.8(3)	Si2[7]-P3-Ba1/Sr1[5]	161.1(3)
Si2-P3-Ba1/Sr1	85.8(2)	Si2-P3-Ba1/Sr1	85.71(14)	Si2-P3-Ba1/Sr1	85.35(13)
Si2-P3-Si2[24]	107.1(4)	Si2[20]-P3-Si2	106.5(2)	Si2-P3-Si2[7]	105.7(3)
Ba1/Sr1-S1-Ba1/Sr1[2]	180.0	Ba1/Sr1[1]-Se1-Ba1/Sr1[2]	180.0	Ba1/Sr1[1]-Te1- Ba1/Sr1[2]	180.0
Ba1/Sr1-S1-Ba1/Sr1[7]	90.0	Ba1/Sr1[5]-Se1- Ba1/Sr1	90.0	Ba1/Sr1[1]-Te1- Ba1/Sr1	90.0
P1[2]-Ba1/Sr1-P1[3]	70.1(2)	P1[8]-Ba1/Sr1-P1[7]	70.9(2)	P1[6]-Ba1/Sr1-P1[7]	72.6(2)
P1[3]-Ba1/Sr1-P1	70.6(3)	P1[7]-Ba1/Sr1-P1	70.8(2)	P1-Ba1/Sr1-P1[7]	71.8(2)
P1[2]-Ba1/Sr1-P1	109.12(8)	P1[8]-Ba1/Sr1-P1	110.14(4)	P1[8]-Ba1/Sr1-P1[7]	112.86(4)
P1-Ba1/Sr1-S1	125.44(4)	P1-Ba1/Sr1-Se1	124.93(2)	P1-Ba1/Sr1-Te1	123.57(2)
S1-Ba1/Sr1-Ba2	180.0	Se1-Ba1/Sr1-Ba2	180.0	Te1-Ba1/Sr1-Ba2	180.0
P1-Ba1/Sr1-Ba2	54.56(4)	P1-Ba1/Sr1-Ba2	55.07(2)	P1-Ba1/Sr1-Ba2	56.43(2)
P1[3]-Ba2-P1[22]	90.0	P1[9]-Ba2-P1[15]	90.0	P1[7]-Ba2-P1[15]	90.0
P1[3]-Ba2-P1[21]	120.0	P1[7]-Ba2-P1[10]	120.0	P1[2]-Ba2-P1[22]	120.0
P1-Si1-P1[12]	104.6(6)	P1-Si1-P1[19]	104.6(3)	P1-Si1-P1[13]	102.7(3)
P1-Si1-P2	108.20(19)	P1-Si1-P2	108.40(12)	P1-Si1-P2	109.32(9)
P2-Si1-P2[13]	118.6(8)	P2-Si1-P2[19]	117.9(5)	P2[13]-Si1-P2	116.0(5)
P1-Si1-Si2	52.3(3)	P1-Si1-Si2	52.28(16)	P1-Si1-Si2	51.35(17)
P2-Si1-Si2	120.7(4)	P2-Si1-Si2	121.1(2)	P2-Si1-Si2	122.0(2)
P1-Si2-P1[12]	104.2(5)	P1-Si2-P1[19]	104.1(3)	P1-Si2-P1[13]	104.5(3)
P1-Si2-P3	109.6(2)	P1-Si2-P3	109.29(1)	P1-Si2-P3	108.76(1)
P3-Si2-P3[14]	114.0(7)	P3-Si2-P3[19]	115.0(4)	P3-Si2-P3[13]	116.6(5)
P1-Si2-Si1	52.1(2)	P1-Si2-Si1	52.04(16)	P1-Si2-Si1	52.24(16)
P3-Si2-Si1	123.0(4)	P3-Si2-Si1	122.5(2)	P3-Si2-Si1	121.7(2)
		**$Sr[Sr_6Se][Si_{12}P_{20}]$**		**$Sr[Sr_6Te][Si_{12}P_{20}]$**	
		Si1[12]-P1-Si1	75.55(8)	Si1[14]-P1-Si1	76.18(6)
		Si1-P1-Sr1	98.50(2)	Si1-P1-Sr1[7]	99.43(16)

Si1-P2-Si1[13]	105.79(6)	Si1-P2-Si1[10]	105.72(5)
Sr1[1]-Se1-Sr1[2]	180.0	Sr1[1]-Te1-Sr1	180.0
Sr1[4]-Se1-Sr1	90.0	Sr1[4]-Te1-Sr1	90.0
Sr1[1]- Sr1- Sr1[2]	60.0	Sr1[3]- Sr1- Sr1[2]	60.0
Sr1[4]- Sr1- Sr1[2]	90.0	Sr1[3]- Sr1- Sr1[4]	90.0
P1-Sr1-P19	71.55(3)	P1-Sr1-P1[10]	72.76(2)
P1[6]-Sr1-P2[8]	133.77(19)	P1[9]-Sr1-P2[6]	134.68(14)
P1-Sr1-Se1	124.23(3)	P1-Sr1-Te1	122.99(2)
Se1-Sr1-Sr1[2]	45.0	Te1-Ba1-Ba1[2]	45.0
Sr1[9]-Sr2-Sr1[18]	180.0	Sr1[17]-Sr2-Sr1	180.0
Sr1-Sr2-Sr1[19]	90.0	Sr1[9]-Sr2-Sr1:	90.0
P1[12]-Si1-P1	104.45(8)	P1[16]-Si1-P1	103.82(6)
P1-Si1-P2	108.83(3	P1-Si1-P2[13]	108.94(2)
P2-Si1-P2[14]	116.41(11)	P2[15]-Si1-P2[13]	116.53(8)
P1-Si1-Si1[12]	52.23(4)	Si1[14]-Si1-P1	51.91(3)
P2-Si1-Si1[12]	121.79(6)	Si1[14]-Si1-P2[13]	121.74(4)

Table S5. Anisotropic Displacement Parameters (Å^2×10^3).
The Anisotropic displacement factor exponent takes the form: -2π^2[h^{2}a*^{2}U$_{11}$+2hka*b*U$_{12}$+…].

Atom	U_{11}	U_{22}	U_{33}	U_{23}	U_{13}	U_{12}
			**Ba[Ba$_6$S][Si$_{12}$P$_{20}$]**			
Ba1	11.99(16)	14.94(12)	14.94(12)	0	0	0
Ba2	70.8(4)	70.8(4)	70.8(4)	0	0	0
Si1	10.1(2)	10.1(2)	7.4(4)	0	0	0.4(3)
P1	11.8(2)	11.8(2)	10.0(4)	0	0	-2.1(3)
P2	9.5(2)	9.5(2)	9.5(2)	0.48(19)	-0.48(19)	-0.48(19)
S1	12.1(5)	12.1(5)	12.1(5)	0	0	0
			**Ba[Ba$_6$Se][Si$_{12}$P$_{20}$]**			
Ba1	16.6(3)	16.6(3)	12.5(4)	0	0	0

Ba2	84.5(12)	84.5(12)	84.5(12)	0	0	0
Si1	11.8(6)	8.3(10)	11.8(6)	0	0.2(7)	0
P1	11.1(6)	11.6(9)	11.1(6)	0	2.6(7)	0
P2	10.3(5)	10.3(5)	10.3(5)	-0.3(5)	0.3(5)	-0.3(5)
Se1	13.2(6)	13.2(6)	13.2(6)	0	0	0
**Ba[Ba_6Te][$Si_{12}P_{20}$]**						
Ba1	17.79(16)	17.79(16)	13.2(2)	0	0	0
Ba2	115.1(10)	115.1(10)	115.1(10)	0	0	0
Si1	10.8(3)	7.8(5)	10.8(3)	0	-0.1(4)	0
P1	12.3(3)	10.0(5)	12.3(3)	0	2.2(4)	0
P2	10.4(3)	10.4(3)	10.4(3)	-0.5(3)	0.5(3)	-0.5(3)
Te1	16.7(2)	16.7(2)	16.7(2)	0	0	0
**Ba[Ba_2Sr_4S][$Si_{12}P_{20}$]**						
Ba1/Sr1	25.5(7)	21.0(5)	21.0(5)	2(2)	0	0
Ba2	63.9(9)	63.9(9)	63.9(9)	0	0	0
Si1	12(3)	15(6)	12(3)	0	-5(4)	0
Si2	14(3)	4(5)	14(3)	0	3(4)	0
P1	16.4(8)	11.0(12)	16.4(8)	-4(3)	-4.2(9)	-4(3)
P2	7(2)	7(2)	7(2)	2.1(19)	-2.1(19)	-2.1(19)
P3	15(3)	15(3)	15(3)	5(2)	5(2)	-5(2)
S1	14.4(15)	14.4(15)	14.4(15)	0	0	0
**Ba[Ba_2Sr_4Se][$Si_{12}P_{20}$]**						
Ba1/Sr1	19.3(3)	15.6(3)	19.3(3)	0	-0.2(12)	0
Ba2	51.5(4)	51.5(4)	51.5(4)	0	0	0
Si1	7.4(18)	7.4(18)	7(3)	0	0	-3(2)
Si2	14(2)	14(2)	7(3)	0	0	2(3)
P1	12.8(4)	12.8(4)	9.5(6)	-4.6(18)	-4.6(18)	-3.6(4)
P2	9.9(17)	9.9(17)	9.9(17)	-2.8(13)	2.8(13)	-2.8(13)
P3	9.1(18)	9.1(18)	9.1(18)	-2.9(12)	2.9(12)	2.9(12)
Se1	19.2(4)	19.2(4)	19.2(4)	0	0	0

**$Ba[Ba_2Sr_4Te][Si_{12}P_{20}]$**						
Ba1/Sr1	17.4(3)	21.3(3)	21.3(3)	4.9(12)	0	0
Ba2	59.7(5)	59.7(5)	59.7(5)	0	0	0
Si1	11.9(17)	11.9(17)	10(3)	0	0	-7(3)
Si2	8.0(16)	8.0(16)	2(3)	0	0	4(2)
P1	12.7(4)	12.7(4)	7.7(6)	2.4(17)	2.4(17)	-4.9(4)
P2	9.0(14)	9.0(14)	9.0(14)	0.2(17)	-0.2(17)	0.2(17)
P3	8.8(14)	8.8(14)	8.8(14)	0.3(18)	-0.3(18)	-0.3(18)
Te1	22.7(3)	22.7(3)	22.7(3)	0	0	0
**$Sr[Sr_6Se][Si_{12}P_{20}]$**						
Sr1	35.7(4)	38.6(5)	35.7(4)	0	0	0
Sr2	235(5)	235(5)	235(5)	0	0	0
Si1	26.6(5)	26.6(5)	22.2(7)	0	0	-0.7(6)
P1	28.9(5)	28.9(5)	24.1(7)	0	0	-3.6(6)
P2	25.9(4)	25.9(4)	25.9(4)	-0.2(4)	0.2(4)	-0.2(4)
Se1	27.8(5)	27.8(5)	27.8(5)	0	0	0
**$Sr[Sr_6Te][Si_{12}P_{20}]$**						
Sr1	23.3(3)	20.1(4)	23.3(3)	-0	-0	0
Sr2	189(4)	189(4)	189(4)	-0	-0	0
Si1	8.4(6)	13.8(4)	13.8(4)	-0	-0	-0.1(5)
P1	11.0(6)	15.8(4)	15.8(4)	-0	-0	-2.5(5)
P2	12.8(3)	12.8(3)	12.8(3)	-0.3(4)	0.3(4)	-0.3(4)
Te1	16.1(3)	16.1(3)	16.1(3)	-0	-0	0

Table S6. Crystallographic information of compounds containing six-coordinated $[A_6Q]/[A_6X]$ ionic group.

Compounds	Space Group	Symmetry	Cell Parameters	Reference
$[(Cs_6F)(Cs_3AgF)][Ge_{14}O_{32}]$	$F\bar{4}3m$	NCS	15.51 15.51 15.51 90 90 90	19
$[(Cs_6F)(Cs_3AgF)][Ge_{12}Mn_2O_{32}]$	$F\bar{4}3m$	NCS	15.52 15.52 15.52 90 90 90	20
$[(Rb_6F)(Rb_4F)][Ge_{14}O_{32}]$	$F\bar{4}3m$	NCS	15.35 15.35 15.35 90 90 90	21
$[(Rb_6F)(Rb_{3.1}Co_{0.9}F_{0.96})][Co_{3.8}Ge_{10.2}O_{30}F_2]$	$F\bar{4}3m$	NCS	15.27 15.27 15.27 90 90 90	21
$Na[K_6F][(UO_2)_3(Si_2O_7)_2]$	$Pnnm$	CS	11.08 12.11 7.84 90 90 90	22
$K[K_6Cl][(UO_2)_3(Si_2O_7)_2]$	$Pnnm$	CS	11.08 13.58 7.87 90 90 90	22
$Na[Rb_6F][(UO_2)_3(Si_2O_7)_2]$	$Pnnm$	CS	11.14 13.51 7.89 90 90 90	22
$K[K_6Cl][(UO_2)_3(Ge_2O_7)_2]$	$Pnnm$	CS	11.38 13.72 8.05 90 90 90	23
$K[K_6Br_{0.6}F_{0.4}][(UO_2)_3(Ge_2O_7)_2]$	$Pnnm$	CS	11.39 13.76 8.06 90 90 90	23
$Na_{0.9}Rb_{0.1}[Rb_6F][(UO_2)_3(Ge_2O_7)_2]$	$Pnnm$	CS	11.40 13.67 8.09 90 90 90	23
$K_{0.6}Na_{0.4}[K_5CsCl_{0.5}F_{0.5}][(UO_2)_3(Ge_2O_7)_2]$	$Pnnm$	CS	11.40 13.77 8.01 90 90 90	23
$K_{0.8}Na_{0.2}[K_{4.8}Cs_{1.2}Cl_{0.5}F_{0.5}][(UO_2)_3(Ge_2O_7)_2]$	$Pmn2_1$	CS	11.43 13.83 7.99 90 90 90	23
$K[K_{1.8}Cs_{4.2}F][(UO_2)_3(Ge_2O_7)_2]$	$Cmc2_1$	CS	22.88 14.28 7.99 90 90 90	23
$[Cs_6Cl][Ga_5GeS_{12}]$	$R\bar{3}m$	CS	11.47 11.47 20.04 90 90 120	24
$[Cs_6Cl][Ga_5GeSe_{12}]$	$R\bar{3}m$	CS	11.87 11.87 20.38 90 90 120	24
$Na[Cs_6F][Ga_6S_{12}]$	$R\bar{3}m$	CS	11.38 11.38 19.29 90 90 120	25
$Na[Cs_6Cl][Ga_6S_{12}]$	$R\bar{3}m$	CS	11.49 11.49 19.74 90 90 120	25
$K[Cs_{5.57}K_{0.43}Cl][Ga_6S_{12}]$	$R\bar{3}m$	CS	11.62 11.62 19.90 90 90 120	25
$Rb[Cs_{5.39}Rb_{0.61}Cl][Ga_6S_{12}]$	$R\bar{3}m$	CS	11.73 11.73 20.00 90 90 120	25
$Na[Cs_{5.69}Na_{1.31}Br][Ga_6S_{12}]$	$R\bar{3}m$	CS	11.51 11.51 19.86 90 90 120	25
$K[Cs_{5.23}K_{1.77}Br][Ga_6S_{12}]$	$R\bar{3}m$	CS	11.64 11.64 19.97 90 90 120	25
$Rb[Cs_{4.92}Rb_{1.08}Br][Ga_6S_{12}]$	$R\bar{3}m$	CS	11.76 11.76 20.10 90 90 120	25
$Na[Cs_6Cl][Ga_6Se_{12}]$	$R\bar{3}m$	CS	11.93 11.93 20.23 90 90 120	25
$K[Cs_{5.74}K_{0.26}Cl][Ga_6Se_{12}]$	$R\bar{3}m$	CS	12.07 12.07 20.47 90 90 120	25
$Na[Cs_6Br][Ga_6Se_{12}]$	$R\bar{3}m$	CS	11.97 11.97 20.40 90 90 120	25
$Rb[Cs_{4.48}Rb_{0.52}Br][Ga_6Se_{12}]$	$R\bar{3}m$	CS	11.93 11.93 20.22 90 90 120	25
$K[Cs_{5.58}K_{0.42}Br][Ga_6Se_{12}]$	$R\bar{3}m$	CS	12.11 12.11 20.61 90 90 120	25
$[Cs_6Cl][Ho_{21}S_{34}]$	$C2/m$	CS	17.13 19.49 12.99 90 128.67 90	26

$[Cs_6Cl][Ho_{21}Se_{34}]$	*C*2/*m*	CS	17.67 20.18 13.42 90 128.61 90	26
$[Cs_6Cl][Ho_{21}Te_{34}]$	*C*2/*m*	CS	18.78 21.49 14.30 90 128.63 90	26
$[Cs_6Cl][Dy_{21}Se_{34}]$	*C*2/*m*	CS	17.17 19.53 13.02 90 128.74 90	26
$[Cs_6Cl][Dy_{21}Te_{34}]$	*C*2/*m*	CS	18.87 21.58 14.36 90 128.66 90	26
$Cs_3[Cs_6Cl]_6[Ga_{53}Se_{96}]$	$R\bar{3}m$	CS	11.99 11.99 50.01 90 90 120	27
$Cs_5[Cs_6Cl]_2[Ga_{15}Ge_9Se_{48}]$	*I*4/*m*	CS	14.39 14.39 28.01 90 90 90	28
$Cs_5[Cs_6Br]_2[Ga_{15}Ge_9Se_{48}]$	*I*4/*m*	CS	14.38 14.38 27.93 90 90 90	28
$Cs_5[Cs_6I_{0.6}Cl_{0.4}]_2[Ga_{15}Ge_9Se_{48}]$	*I*4/*m*	CS	14.43 14.43 28.08 90 90 90	28
$Cs_5[Rb_6Cl]_2[Ga_{15}Ge_9Se_{48}]$	*I*4/*m*	CS	14.22 14.22 27.41 90 90 90	28
$Cs_5[Cs_5KCl]_2[Ga_{15}Ge_9Se_{48}]$	*I*4/*m*	CS	14.33 14.33 27.80 90 90 90	28
$Cs_5[Rb_6Rb]_2[Ga_{15}Ge_9Se_{48}]$	*I*4/*m*	CS	14.30 14.30 27.60 90 90 90	28
$Li[K_6Cl][Fe_{24}S_{26}]$	$Pm\bar{3}m$	CS	10.36 10.36 10.36 90 90 90	29
$[Cs_6Cl][Fe_{24}Se_{26}]$	*I*4/*mmm*	CS	11.09 11.09 22.14 90 90 90	30
$Fe[Ba_6S][Cu_{12}Fe_{12}S_{26}]$	$Pm\bar{3}m$	CS	10.38 10.38 10.38 90 90 90	31
$Cs_5[Cs_6O][Fe_5S_8]_2$	*I*4/*mmm*	CS	11.99 11.99 14.11 90 90 90	32
$Cs[Ba_6Cl][Si_{12}P_{20}]$	$Fm\bar{3}m$	CS	15.78 15.78 15.78 90 90 90	33
$Cs[Ba_6Br][Si_{12}P_{20}]$	$Fm\bar{3}m$	CS	15.82 15.82 15.82 90 90 90	33
$Cs[Ba_6I][Si_{12}P_{20}]$	$Fm\bar{3}m$	CS	15.86 15.86 15.86 90 90 90	33
$Rb[Ba_6Cl][Si_{12}P_{20}]$	$Fm\bar{3}m$	CS	15.76 15.76 15.76 90 90 90	33
$Rb[Ba_6Br][Si_{12}P_{20}]$	$Fm\bar{3}m$	CS	15.80 15.80 15.80 90 90 90	33
$Rb[Ba_6I][Si_{12}P_{20}]$	$Fm\bar{3}m$	CS	15.86 15.86 15.86 90 90 90	33
$K[Ba_6Cl][Si_{12}P_{20}]$	$Fm\bar{3}m$	CS	15.73 15.73 15.73 90 90 90	33
$Na[Ba_6Cl][Si_{12}P_{20}]$	$Fm\bar{3}m$	CS	15.67 15.67 15.67 90 90 90	33
$Cs[Sr_6Cl][Si_{12}P_{20}]$	$Fm\bar{3}m$	CS	15.64 15.64 15.64 90 90 90	33
$Cs[Sr_6Br][Si_{12}P_{20}]$	$Fm\bar{3}m$	CS	15.67 15.67 15.67 90 90 90	33
$Rb[Sr_6Br][Si_{12}P_{20}]$	$Fm\bar{3}m$	CS	15.63 15.63 15.63 90 90 90	33
$[Ba_4(Ba_6Cl_2)][(VO_4)_6]$	$P6_3/m$	CS	10.56 10.56 7.76 90 90 120	34
$Ba_4[Ba_6S][(VO_3S)_6]$	$P6_3$	NCS	18.41 18.41 8.63 90 90 120	35
$Ba_4[Ba_6S][(V_6O_{2.78}S_{1.22})_6]$	$P6_3$	NCS	18.32 18.32 8.60 90 90 120	35

Table S7. Average coordination number (ACN) of Ba/Sr-Si-P and A-M-Pn-X series compounds. (Calculated by the covalent coordination number of P atom in a single cell, the coplanar weight of P atom is 0.5, the common edge weight is 0.25, and the common vertex weight is 0.125.)

Compounds	Band gap (eV)	ACN	Calculation process (The red is the covalent coordination number of P atom)
$A[A_6Ch][Si_{12}P_{20}]$	1.91-2.27	2.40	(24x2+48x0.5x2+32x3)/24+48x0.5+32=192/80
$BaSi_7P_{10}$ [36]	1.48	2.80	(2x2+8x0.125x3+7x3)/2+8x0.125+1=28/10
$SrSi_7P_{10}$ [36]	1.51		
$Ba_2Si_3P_6$ (exist P-P bond) [37]	1.88	2.42	(13x2+4x0.5x2+4x0.25x2+6x3+4x0.5x3)/ (13+4x0.5+4x0.25+6+4x0.5)=58/24
Ba_2SiP_4 (exist P-P bond) [38]	1.45	2.00	(16x2)/16=32/16
Sr_2SiP_4 (exist P-P bond) [38]	1.41		
$[Sr_4Br]_2[Mg_3Si_{25}P_{40}]$[39]	1.90	2.8	(8x2+32x3)/40=112/40
$[Sr_4Br]_2[Cd_3Si_{25}P_{40}]$ [39]	1.83		
$[Ba_3Br][GaSi_{10}P_{16}]$ [39]	1.86	2.75	(16x2+46x3+4x0.5x3)/(16+46+4x0.5)=176/64
$[Ba_3Br][InSi_{10}P_{16}]$ [39]	1.81		
$[Sr_4Br][In_2Si_{11}P_{19}]$ [40]	1.87	2.68	(5x2+1x0.5x2+10x3+3x0.5x3+1x4)/(5+0.5x2+ 10+3x0.5+1)=49.5/18.5
$[Ba_2Sr_4Br_2][Sr_4Br][In_5Si_{31}P_{52}]$ [40]	1.70	2.77	(24x2+80x3)/104=288/104

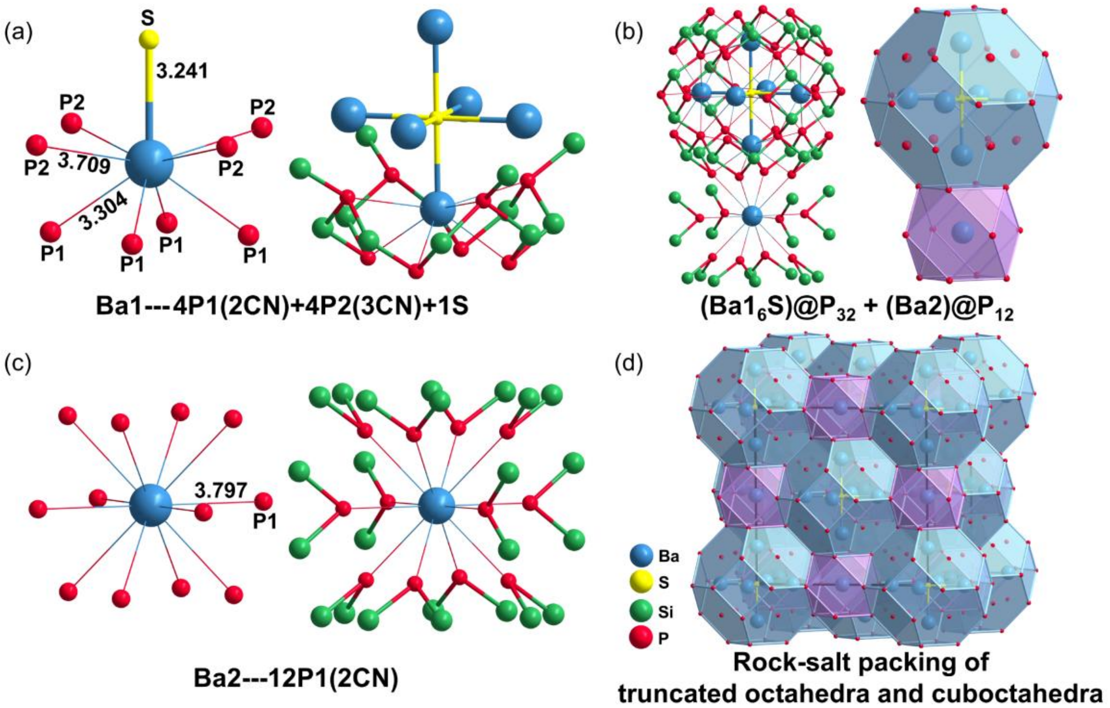

Figure S1. Coordination environments of Ba1 and Ba2 atoms in $Ba[Ba_6S][Si_{12}P_{20}]$. (a) Coordination environment of Ba1. (b) Coordination environment of Ba2. (c) Truncated octahedron $(Ba1_6S)@P_{32}$ and cuboctahedron $(Ba2)@P_{12}$. (d) Rock-salt sublattice packing in $Ba[Ba_6S][Si_{12}P_{20}]$.

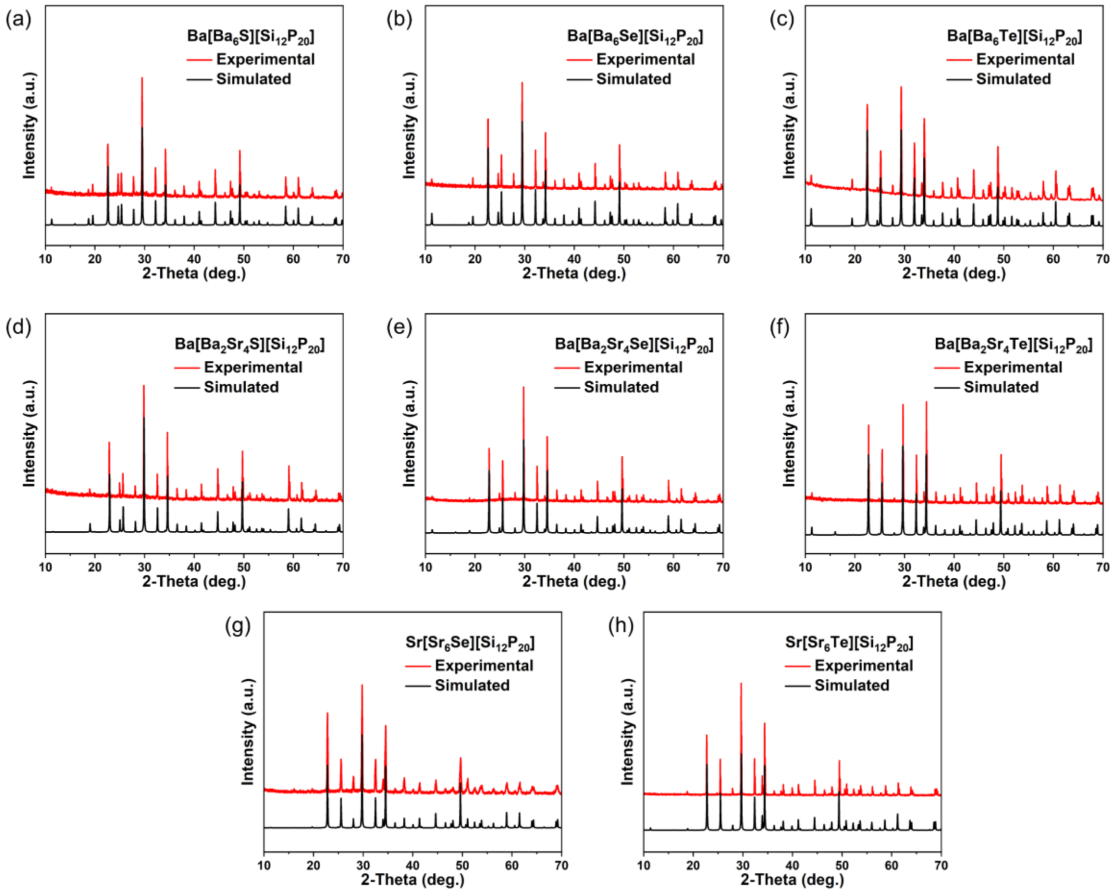

Figure S2. Powder XRD patterns. Powder XRD patterns of the experimental and simulated for $Ba[Ba_6S][Si_{12}P_{20}]$ (a), $Ba[Ba_6Se][Si_{12}P_{20}]$ (b), $Ba[Ba_6Te][Si_{12}P_{20}]$ (c), $Ba[Ba_2Sr_4S][Si_{12}P_{20}]$ (d), $Ba[Ba_2Sr_4Se][Si_{12}P_{20}]$ (e), $Ba[Ba_2Sr_4Te][Si_{12}P_{20}]$ (f), $Sr[Sr_6Se][Si_{12}P_{20}]$ (g) and $Sr[Sr_6Te][Si_{12}P_{20}]$ (h).

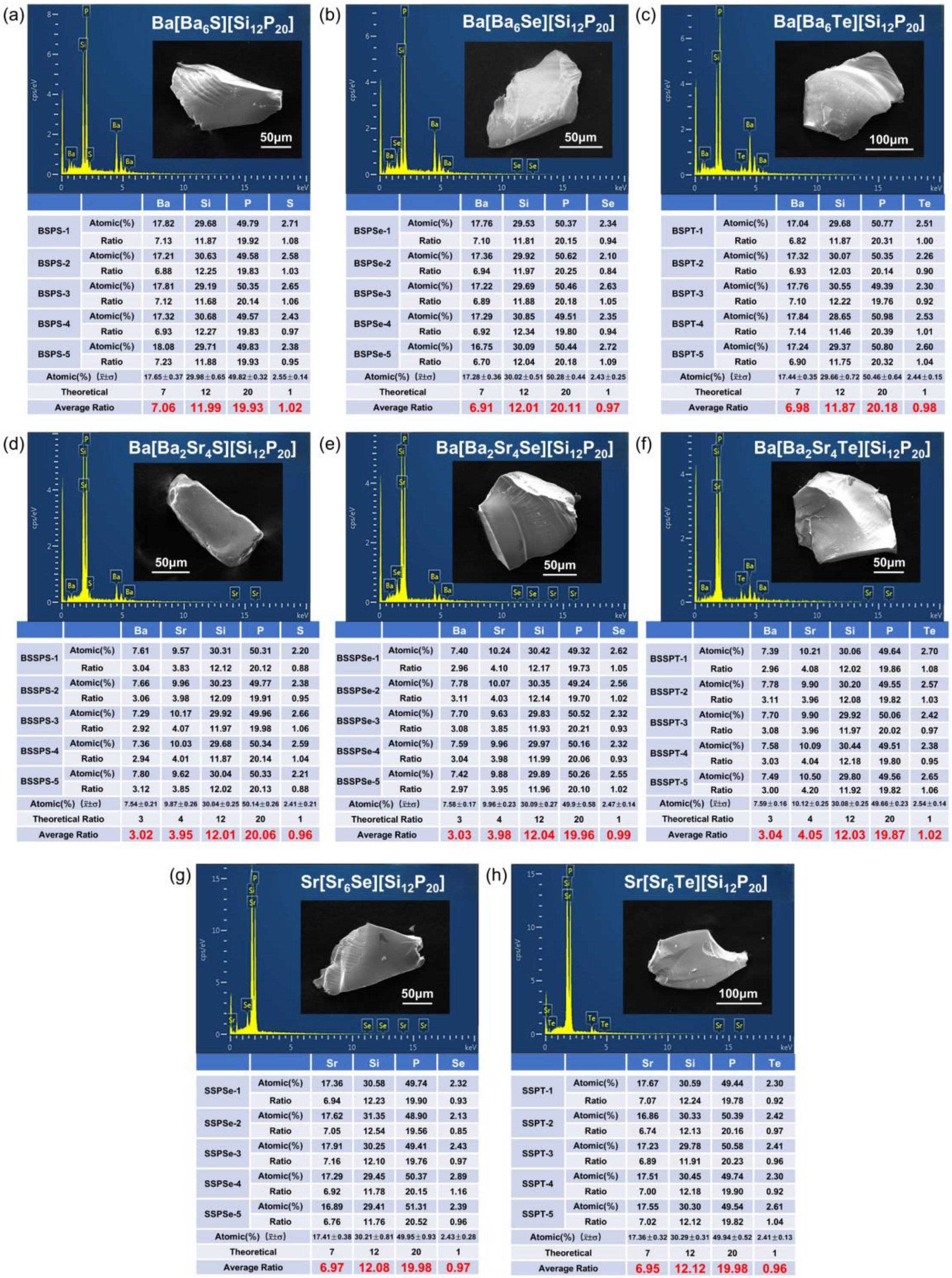

(a)

		Ba	Si	P	S
BSPS-1	Atomic(%)	17.82	29.68	49.79	2.71
	Ratio	7.13	11.87	19.92	1.08
BSPS-2	Atomic(%)	17.21	30.63	49.58	2.58
	Ratio	6.88	12.25	19.83	1.03
BSPS-3	Atomic(%)	17.81	29.19	50.35	2.65
	Ratio	7.12	11.68	20.14	1.06
BSPS-4	Atomic(%)	17.32	30.68	49.57	2.43
	Ratio	6.93	12.27	19.83	0.97
BSPS-5	Atomic(%)	18.08	29.71	49.83	2.38
	Ratio	7.23	11.88	19.93	0.95
Atomic(%) ($\bar{x}\pm\sigma$)		17.65±0.37	29.98±0.65	49.82±0.32	2.55±0.14
Theoretical		7	12	20	1
Average Ratio		7.06	11.99	19.93	1.02

(b)

		Ba	Si	P	Se
BSPSe-1	Atomic(%)	17.76	29.53	50.37	2.34
	Ratio	7.10	11.81	20.15	0.94
BSPSe-2	Atomic(%)	17.36	29.92	50.62	2.10
	Ratio	6.94	11.97	20.25	0.84
BSPSe-3	Atomic(%)	17.22	29.69	50.46	2.63
	Ratio	6.89	11.88	20.18	1.05
BSPSe-4	Atomic(%)	17.29	30.85	49.51	2.35
	Ratio	6.92	12.34	19.80	0.94
BSPSe-5	Atomic(%)	16.75	30.09	50.44	2.72
	Ratio	6.70	12.04	20.18	1.09
Atomic(%) ($\bar{x}\pm\sigma$)		17.28±0.36	30.02±0.51	50.28±0.44	2.43±0.25
Theoretical		7	12	20	1
Average Ratio		6.91	12.01	20.11	0.97

(c)

		Ba	Si	P	Te
BSPT-1	Atomic(%)	17.04	29.68	50.77	2.51
	Ratio	6.82	11.87	20.31	1.00
BSPT-2	Atomic(%)	17.32	30.07	50.35	2.26
	Ratio	6.93	12.03	20.14	0.90
BSPT-3	Atomic(%)	17.76	30.55	49.39	2.30
	Ratio	7.10	12.22	19.76	0.92
BSPT-4	Atomic(%)	17.84	28.65	50.98	2.53
	Ratio	7.14	11.46	20.39	1.01
BSPT-5	Atomic(%)	17.24	29.37	50.80	2.60
	Ratio	6.90	11.75	20.32	1.04
Atomic(%) ($\bar{x}\pm\sigma$)		17.44±0.35	29.66±0.72	50.46±0.64	2.44±0.15
Theoretical		7	12	20	1
Average Ratio		6.98	11.87	20.18	0.98

(d)

		Ba	Sr	Si	P	S
BSSPS-1	Atomic(%)	7.61	9.57	30.31	50.31	2.20
	Ratio	3.04	3.83	12.12	20.12	0.88
BSSPS-2	Atomic(%)	7.66	9.96	30.23	49.77	2.38
	Ratio	3.06	3.98	12.09	19.91	0.95
BSSPS-3	Atomic(%)	7.29	10.17	29.92	49.96	2.66
	Ratio	2.92	4.07	11.97	19.98	1.06
BSSPS-4	Atomic(%)	7.36	10.03	29.68	50.34	2.59
	Ratio	2.94	4.01	11.87	20.14	1.04
BSSPS-5	Atomic(%)	7.80	9.62	30.04	50.33	2.21
	Ratio	3.12	3.85	12.02	20.13	0.88
Atomic(%) ($\bar{x}\pm\sigma$)		7.54±0.21	9.87±0.26	30.04±0.25	50.14±0.26	2.41±0.21
Theoretical Ratio		3	4	12	20	1
Average Ratio		3.02	3.95	12.01	20.06	0.96

(e)

		Ba	Sr	Si	P	Se
BSSPSe-1	Atomic(%)	7.40	10.24	30.42	49.32	2.62
	Ratio	2.96	4.10	12.17	19.73	1.05
BSSPSe-2	Atomic(%)	7.78	10.07	30.35	49.24	2.56
	Ratio	3.11	4.03	12.14	19.70	1.02
BSSPSe-3	Atomic(%)	7.70	9.63	29.83	50.52	2.32
	Ratio	3.08	3.85	11.93	20.21	0.93
BSSPSe-4	Atomic(%)	7.59	9.96	29.97	50.16	2.32
	Ratio	3.04	3.98	11.99	20.06	0.93
BSSPSe-5	Atomic(%)	7.42	9.88	29.89	50.26	2.55
	Ratio	2.97	3.95	11.96	20.10	1.02
Atomic(%) ($\bar{x}\pm\sigma$)		7.58±0.17	9.96±0.23	30.09±0.27	49.9±0.58	2.47±0.14
Theoretical Ratio		3	4	12	20	1
Average Ratio		3.03	3.98	12.04	19.96	0.99

(f)

		Ba	Sr	Si	P	Te
BSSPT-1	Atomic(%)	7.39	10.21	30.06	49.64	2.70
	Ratio	2.96	4.08	12.02	19.86	1.08
BSSPT-2	Atomic(%)	7.78	9.90	30.20	49.55	2.57
	Ratio	3.11	3.96	12.08	19.82	1.03
BSSPT-3	Atomic(%)	7.70	9.90	29.92	50.06	2.42
	Ratio	3.08	3.96	11.97	20.02	0.97
BSSPT-4	Atomic(%)	7.58	10.09	30.44	49.51	2.38
	Ratio	3.03	4.04	12.18	19.80	0.95
BSSPT-5	Atomic(%)	7.49	10.50	29.80	49.56	2.65
	Ratio	3.00	4.20	11.92	19.82	1.06
Atomic(%) ($\bar{x}\pm\sigma$)		7.59±0.16	10.12±0.25	30.08±0.25	49.66±0.23	2.54±0.14
Theoretical Ratio		3	4	12	20	1
Average Ratio		3.04	4.05	12.03	19.87	1.02

(g)

		Sr	Si	P	Se
SSPSe-1	Atomic(%)	17.36	30.58	49.74	2.32
	Ratio	6.94	12.23	19.90	0.93
SSPSe-2	Atomic(%)	17.62	31.35	48.90	2.13
	Ratio	7.05	12.54	19.56	0.85
SSPSe-3	Atomic(%)	17.91	30.25	49.41	2.43
	Ratio	7.16	12.10	19.76	0.97
SSPSe-4	Atomic(%)	17.29	29.45	50.37	2.89
	Ratio	6.92	11.78	20.15	1.16
SSPSe-5	Atomic(%)	16.89	29.41	51.31	2.39
	Ratio	6.76	11.76	20.52	0.96
Atomic(%) ($\bar{x}\pm\sigma$)		17.41±0.38	30.21±0.81	49.95±0.93	2.43±0.28
Theoretical		7	12	20	1
Average Ratio		6.97	12.08	19.98	0.97

(h)

		Sr	Si	P	Te
SSPT-1	Atomic(%)	17.67	30.59	49.44	2.30
	Ratio	7.07	12.24	19.78	0.92
SSPT-2	Atomic(%)	16.86	30.33	50.39	2.42
	Ratio	6.74	12.13	20.16	0.97
SSPT-3	Atomic(%)	17.23	29.78	50.58	2.41
	Ratio	6.89	11.91	20.23	0.96
SSPT-4	Atomic(%)	17.51	30.45	49.74	2.30
	Ratio	7.00	12.18	19.90	0.92
SSPT-5	Atomic(%)	17.55	30.30	49.54	2.61
	Ratio	7.02	12.12	19.82	1.04
Atomic(%) ($\bar{x}\pm\sigma$)		17.36±0.32	30.29±0.31	49.94±0.52	2.41±0.13
Theoretical		7	12	20	1
Average Ratio		6.95	12.12	19.98	0.96

Figure S3. Energy-dispersive X-ray spectroscopy (EDS) analysis. EDS spectra of $Ba[Ba_6S][Si_{12}P_{20}]$ (a), $Ba[Ba_6Se][Si_{12}P_{20}]$ (b), $Ba[Ba_6Te][Si_{12}P_{20}]$ (c), $Ba[Ba_2Sr_4S][Si_{12}P_{20}]$ (d), $Ba[Ba_2Sr_4Se][Si_{12}P_{20}]$ (e), $Ba[Ba_2Sr_4Te][Si_{12}P_{20}]$ (f), $Sr[Sr_6Se][Si_{12}P_{20}]$ (g) and $Sr[Sr_6Te][Si_{12}P_{20}]$ (h).

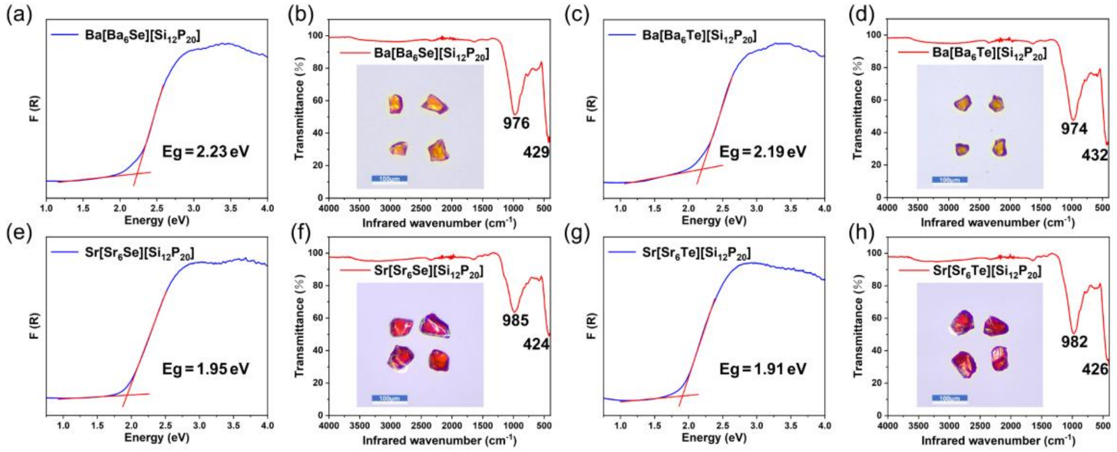

Figure S4. Optical properties. (a, c, e and g) UV-Vis-NIR diffuse reflectance spectra and band gap values. (b, d, f and h) IR ATR transmission spectra and corresponding crystal crystal photos (inset b，d，f and h).

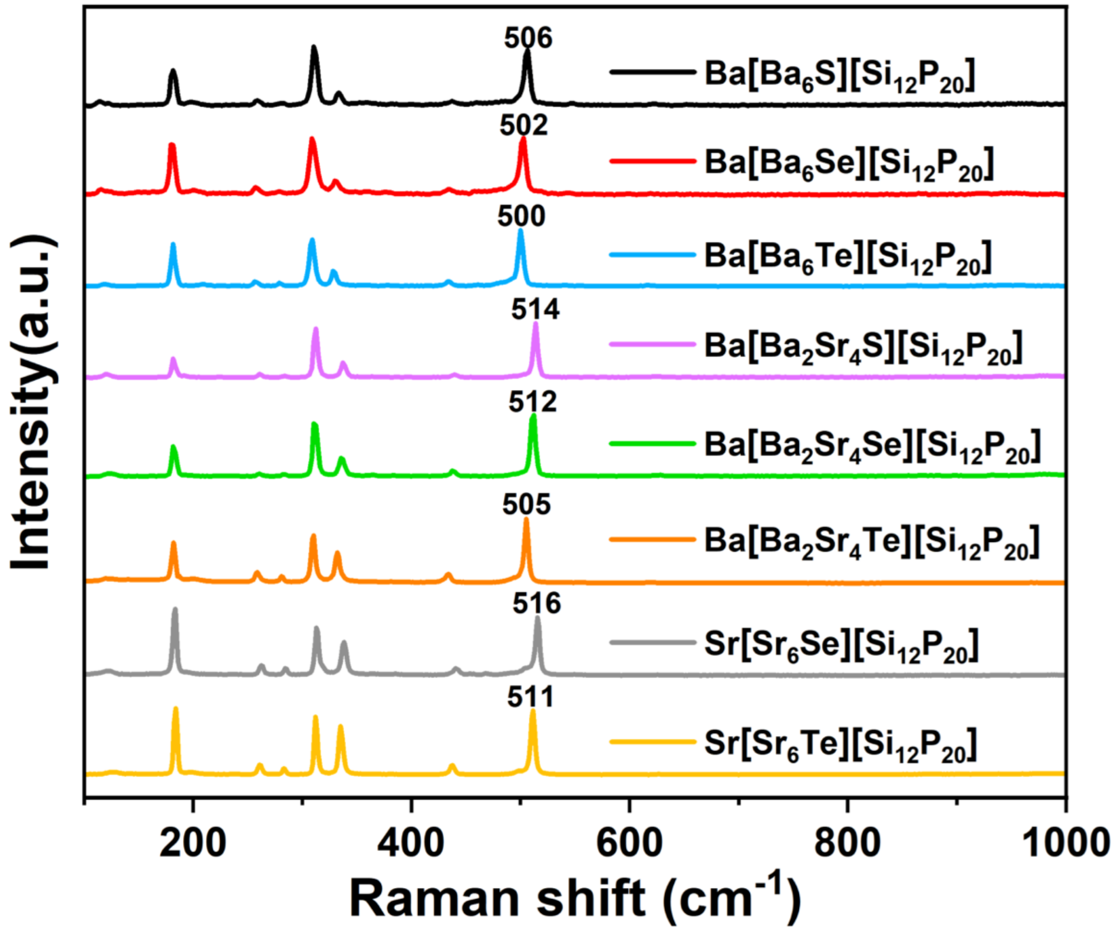

Figure S5. Raman scattering spectra. Raman scattering spectra of Ba[Ba$_6$S][Si$_{12}$P$_{20}$], Ba[Ba$_6$Se][Si$_{12}$P$_{20}$], Ba[Ba$_6$Te][Si$_{12}$P$_{20}$], Ba[Ba$_2$Sr$_4$S][Si$_{12}$P$_{20}$], Ba[Ba$_2$Sr$_4$Se][Si$_{12}$P$_{20}$], Ba[Ba$_2$Sr$_4$Te][Si$_{12}$P$_{20}$], Sr[Sr$_6$Se][Si$_{12}$P$_{20}$] and Sr[Sr$_6$Te][Si$_{12}$P$_{20}$].

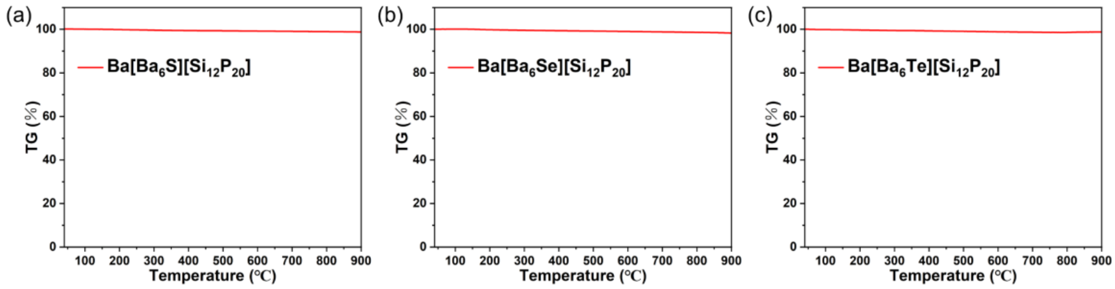

Figure S6. TG curves. TG curves of $Ba[Ba_6S][Si_{12}P_{20}]$ (a), $Ba[Ba_6Se][Si_{12}P_{20}]$ (b) and $Ba[Ba_6Te][Si_{12}P_{20}]$ (c).

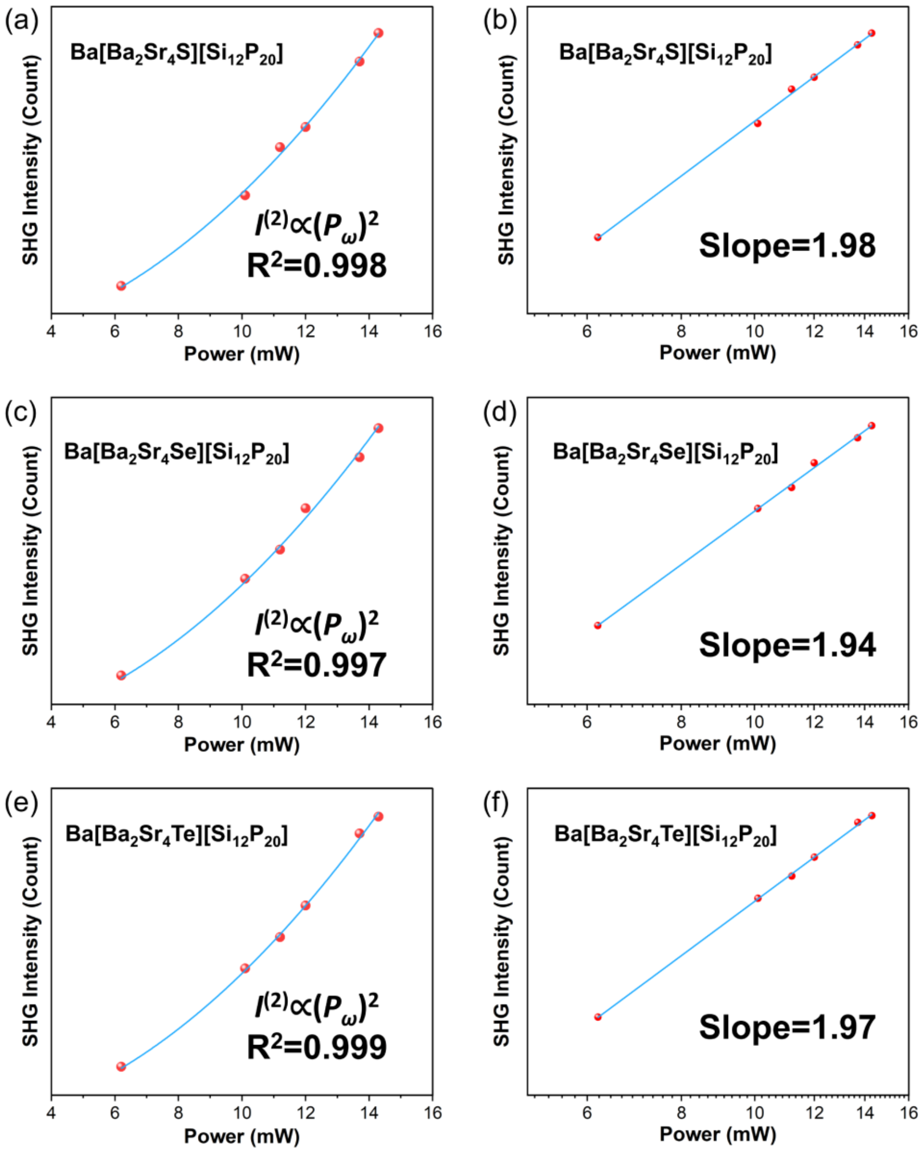

Figure S7. Power-dependent SHG measurements (a, c, e) Linear-scale plots of SHG intensity as a function of incident laser power for $Ba[Ba_2Sr_4S][Si_{12}P_{20}]$, $Ba[Ba_2Sr_4Se][Si_{12}P_{20}]$, and $Ba[Ba_2Sr_4Te][Si_{12}P_{20}]$, respectively; (b, d, f) the corresponding double-logarithmic plots.

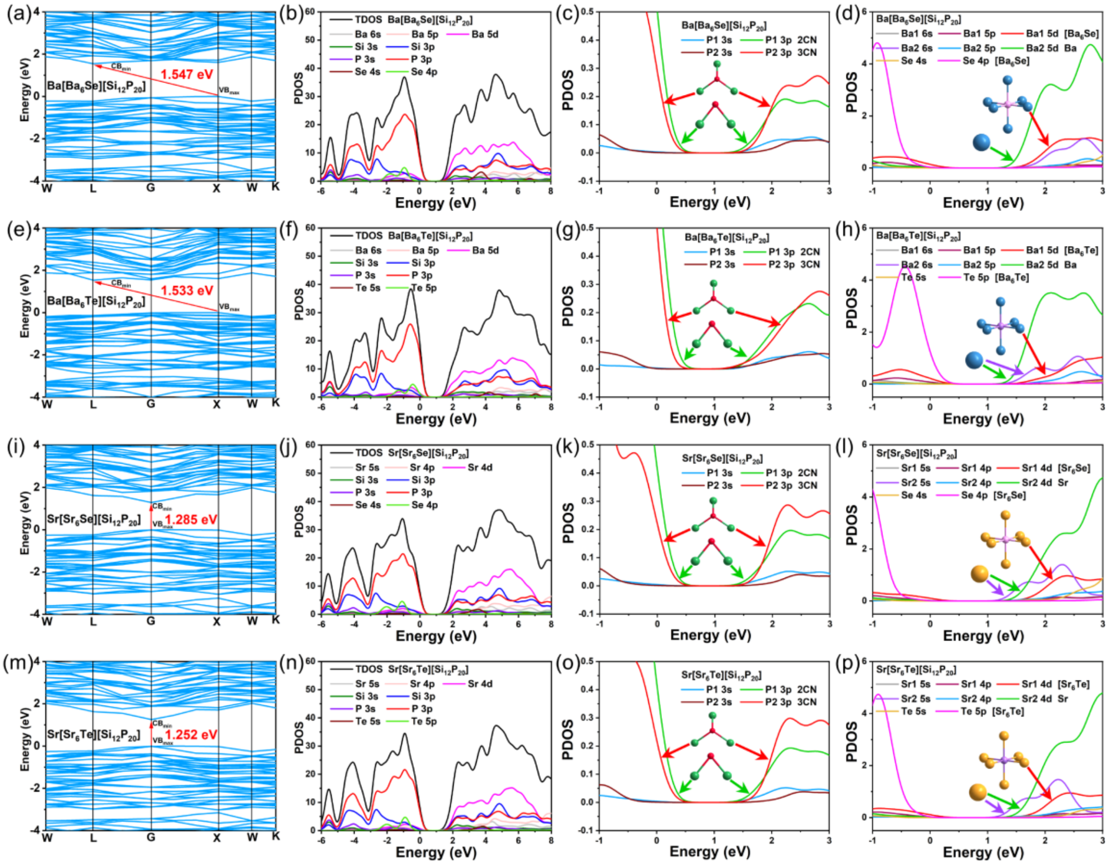

Figure S8. Band structures and PDOS. (a, e, i, m) Band structure diagrams, (b, f, j, n) Density of states (DOS) diagrams, (c, g, k, o) Projected density of states (PDOS) of P1 (2CN), P2/P3 (3CN) atoms. (d, h, l, p) PDOS of Ba and [Ba_6Se], Ba[Ba_6Te], [Sr_6Se], [Sr_6Te] of Ba[Ba_6Se][$Si_{12}P_{20}$], Ba[Ba_6Te][$Si_{12}P_{20}$], Sr[Sr_6Se][$Si_{12}P_{20}$] and Sr[Sr_6Te][$Si_{12}P_{20}$].

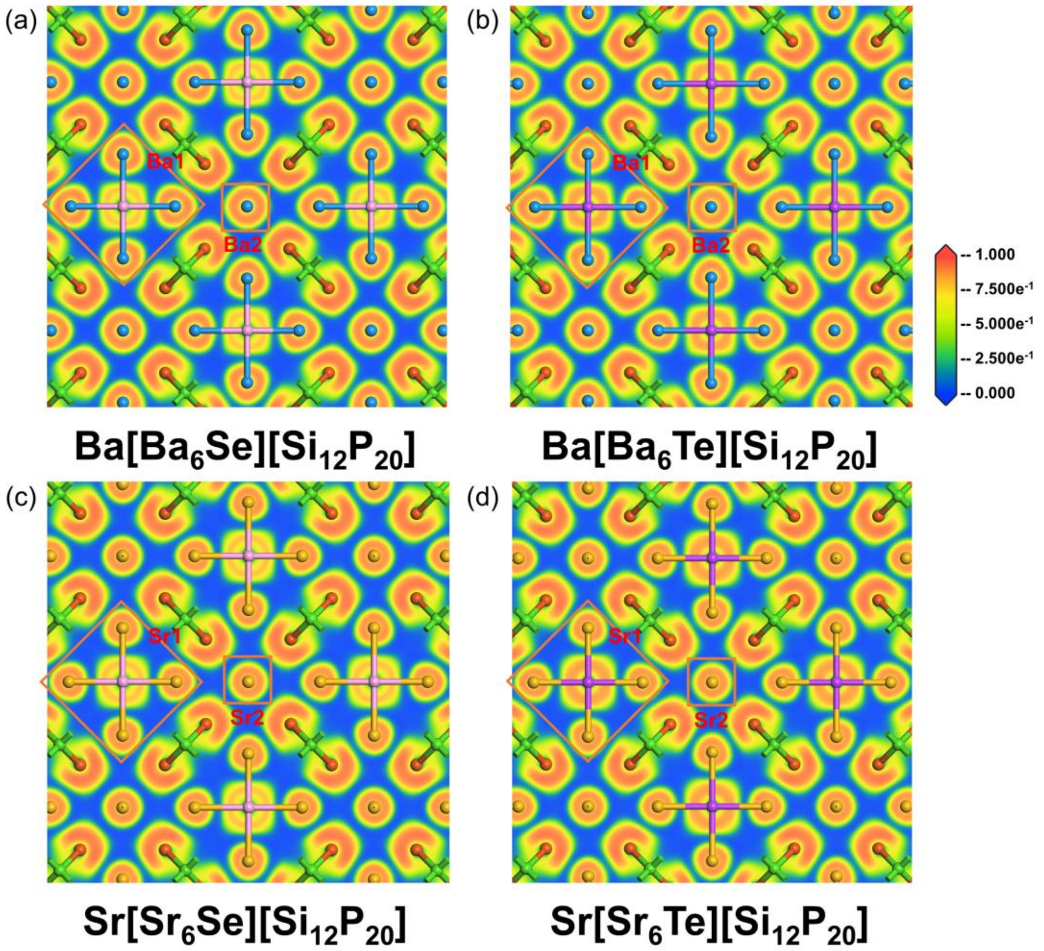

Figure S9. ELF diagrams. Slice electron localization function (ELF) field distribution of $Ba[Ba_6Se][Si_{12}P_{20}]$ (a), $Ba[Ba_6Te][Si_{12}P_{20}]$ (b), $Sr[Sr_6Se][Si_{12}P_{20}]$ (c) and $Sr[Sr_6Te][Si_{12}P_{20}]$ (d).

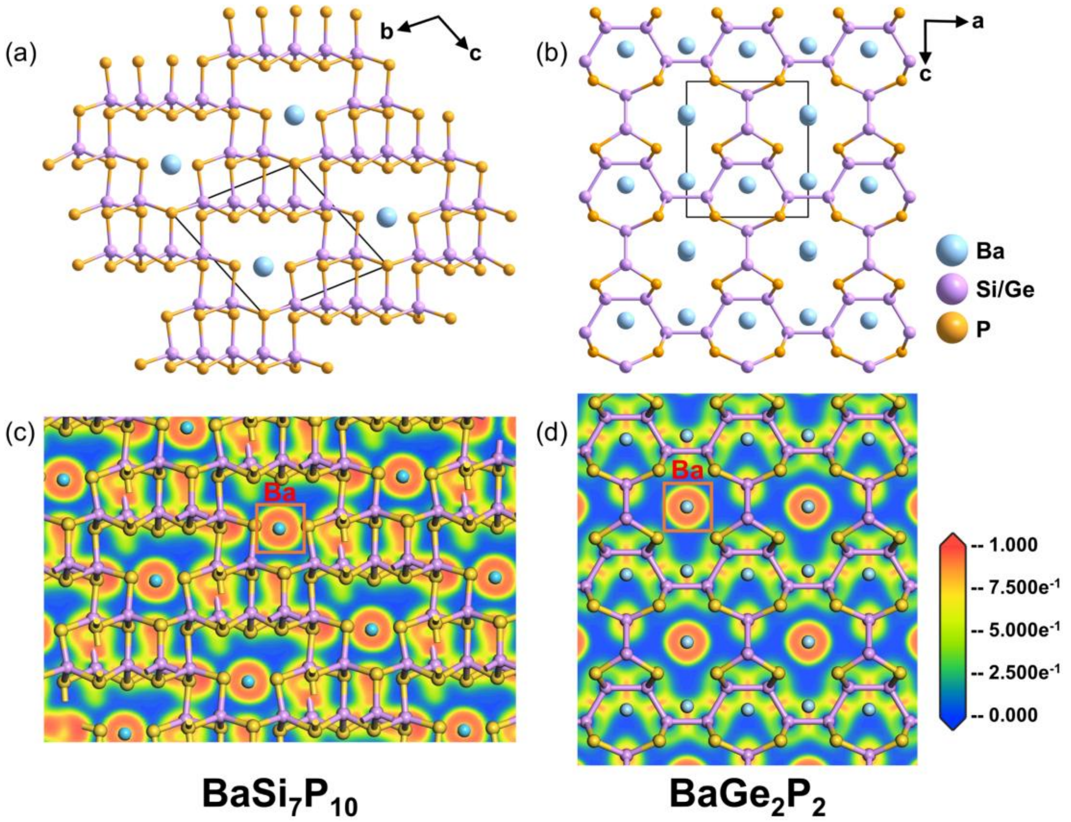

Figure S10. Crystal structure and ELF diagrams. (a, b) Crystal structures of $BaSi_7P_{10}$ and $BaGe_2P_2$; (c, d) corresponding slice ELF field distributions.

References:

[1]. G. M. Sheldrick. *Acta. Cryst*. 2008. A64, 112.

[2]. G. M. Sheldrick, *Cryst. Struct. Chem.* 2015. ***71***, 3-8.

[3]. A. Spek, *J. Appl. Crystallog.* 2003**. 36**, 7-13.

[4]. P. Kubelka and F. Munk, *Z Tech Phys*, 1931, **12**: 593-601.

[5]. S. Kurtz and T. Perry, *J. Appl. Phys.* 1968, **39**, 3798-3813.

[6]. S. J. Clark, M. D. Segall, C. J. Pickard, P. J. Hasnip, M. J. Probert, K. Refson and M. C. Payne, *Z. Kristallogr. Cryst. Mater.* 2005, **220**, 567-570.

[7]. W. Kohn and L. J. Sham, *Phys. Rev.* 1965, **140**, A1133-A1138.

[8]. M. Segall, P. J. Lindan, M. a. Probert, C. J. Pickard, P. J. Hasnip, S. Clark and M. Payne, *J. Phys. Condens. Matter*. 2002, **14**, 2717-2744.

[9]. J. P. Perdew and Y. Wang, *Phys. Rev. B*. 1992, **46**, 12947-12954.

[10]. J. Lin, A. Qteish, M. Payne and V. Heine, *Phys. Rev. B.* 1993, **47**, 4174-4180.

[11]. A. M. Rappe, K. M. Rabe, E. Kaxiras and J. Joannopoulos, 1990, **41**, 1227-1230.

[12]. J. P. Perdew, A. Ruzsinszky, G. I. Csonka, O. A. Vydrov, G. E. Scuseria, L. A. Constantin, X. Zhou and K. Burke, *Phys. Rev. Lett.* 2008, **100**, 136406.

[13]. J. P. Perdew, K. Burke and M. Ernzerhof, *Phys. Rev. Lett.* 1996, **77**, 3865-3868.

[14]. Y. Wang, P. Wisesa, A. Balasubramanian, S. Dwaraknath and T. Mueller, *Comput. Mater. Sci.* 2021, **187**, 110100.

[15]. X. Gonze, *Phys. Rev. A.* 1995, **52**, 1096-1114.

[16]. X. Gonze, *Phys. Rev. A.* 1995, **52**, 1086-1095.
[17]. S. Sharma and C. Ambrosch-Draxl, *Phys. Scr.* 2004, **2004**, 128-134.
[18]. C.-S. Lin, A.-Y. Zhou, W.-D. Cheng, N. Ye and G.-L. Chai, *J. Phys. Chem. C.* 2019, **123**, 31183−31189.
[19]. N. R. Spagnuolo, G. Morrison and H.-C. zur Loye, *Solid. State. Sci*. 2019, **97**, 105973.
[20]. D. Carone, G. Morrison, M. D. Smith and H.-C. zur Loye, *Cryst. Growth. Des.* 2022, **22**, 3319-3325.
[21]. D. Carone, M. Usman, V. V. Klepov, M. D. Smith, V. Kocevski, T. M. Besmann and H.-C. zur Loye, *CrystEngComm*. 2020, **22**, 8072-8080.
[22]. G. Morrison and H.-C. zur Loye, *Cryst. Growth. Des.* 2016, **16**, 1294-1299.
[23]. C. A. Juillerat, E. E. Moore, G. Morrison, M. D. Smith, T. Besmann and H.-C. Zur Loye, *Inorg. Chem.* 2018, **57**, 11606-11615.
[24]. R. Wang, X. Zhang and F. Huang, *Sci. China. Chem.* 2022, **65**, 1903-1910.
[25]. A. A. Berseneva, L. W. Masachchi, L. G. Jacobsohn and H.-C. zur Loye, *Chem. Mater.* 2023, **35**, 1417-1431.
[26]. H. Lin, L.-H. Li and L. Chen, *Inorg. Chem.* 2012, **51**, 4588-4596.
[27]. H. Lin, H. Chen, Z.-X. Lin, H.-J. Zhao, P.-F. Liu, J.-S. Yu and L. Chen, *Inorg. Chem.* 2016, **55**, 1014-1016.
[28]. S. X. Huang ‐ Fu, J. N. Shen, H. Lin, L. Chem. - Eur. J. 2015, **21**, 9809-9815.
[29]. G. Santarini, *Electrochim. Acta*. 1982, **27**, 495-510.
[30]. P. Adler, S. A. Medvedev, M. Valldor, P. G. Naumov, M. A. ElGhazali and R. Rüffer, *Phys. Rev. B.* 2020, **101**, 094433.
[31]. J. Llanos, C. Mujica, O. Wittke, P. Gómez-Romero and R. Ramírez, *J. Solid State Chem.* 1997, **128**, 62-65.
[32]. M. Schwarz and C. Röhr, *Z. Anorg. Allg. Chem*. 2014, **640**, 2792-2800.
[33]. P. Yox, A. P. Porter, R. W. Dorn, V. Kyveryga, A. J. Rossini and K. Kovnir, *Chem. Commun*. 2022, **58**, 7622-7625.
[34]. Y.-H. Roh and S.-T. Hong, *Struct. Rep*. 2005, **61**, i140-i142.
[35]. M.-Y. Ran, S.-H. Zhou, B.-X. Li, X.-T. Wu, H. Lin and Q.-L. Zhu, *Chem. Mater.* 2024, **36**, 11996-12005.
[36]. X. Zhao, C. Lin, J. Chen, F. Xu, S. Yang, G. Peng, H. Tian, Y. Han, B. Li and M. Luo, *Adv. Opt. Mater.* 2022, **10**, 2200045.
[37]. J. Mark, J. Wang, K. Wu, J. G. Lo, S. Lee and K. Kovnir, *J. Am. Chem. Soc.* 2019, **141**, 11976-11983.
[38]. J. Mark, J. A. Dolyniuk, N. Tran and K. Kovnir, *Z. Anorg. Allg. Chem.* 2019, **645**, 242-247.
[39]. L. Gao, J. Chen, X. Shi, Y. Xiao, Y. Han, C. Lin, H. Jiang, G. Yang, G. Peng and N. Ye, *Sci. Adv.* 2024, **10**, eadr2389.
[40]. M.-S. Zhang, S.-M. Pei, B.-W. Liu, X.-M. Jiang and G.-C. Guo, *Sci. China Chem.* 2025, **68**, 4134-4140.
